# Supplementary material for: NT-proBNP testing for heart failure diagnosis in people with atrial fibrillation: A diagnostic accuracy study
Source: PLoS Med. 2025 Oct 30;22(10):e1004550. doi: 10.1371/journal.pmed.1004550 (PMC12574882; doi:10.1371/journal.pmed.1004550)
Supplement: S1 Table — (PDF) [file pmed.1004550.s001.pdf]

Supplementary Table 1. Diagnostic test accuracy parameters for the diagnosis of HF using NT-proBNP level **by age group** at NICE and ESC referral thresholds for those **without** pre-existing atrial fibrillation

	**Age <65 years** (n=87,985)				**Age 65-74 years** (n=36,296)				**Age 75+ years** (n=13,663)			
NT-proBNP threshold (pg/mL)	≥125	≥400	≥660	≥2000	≥125	≥400	≥660	≥2000	≥125	≥400	≥660	≥2000
Prevalence % (95% CI)	5.1 (5-5.3)	5.1 (5-5.3)	5.1 (5-5.3)	5.1 (5-5.3)	10.7 (10.4-11)	10.7 (10.4-11)	10.7 (10.4-11)	10.7 (10.4-11)	14.8 (14.2-15.4)	14.8 (14.2-15.4)	14.8 (14.2-15.4)	14.8 (14.2-15.4)
TP, n	4001	3157	2563	1383	3718	3143	2604	1462	1962	1729	1462	885
FN, n	506	1350	1944	3124	171	746	1285	2427	59	292	559	1136
FP, n	28884	7224	3879	1017	20955	7764	4492	1273	9032	4260	2668	856
TN, n	54594	76254	79599	82461	11452	24643	27915	31134	2610	7382	8974	10786
Sensitivity % (95% CI)	88.8 (87.8-89.7)	70.0 (68.7-71.4)	56.9 (55.4-58.3)	30.7 (29.3-32.1)	95.6 (94.9-96.2)	80.8 (79.5-82)	67.0 (65.5-68.4)	37.6 (36.1-39.1)	97.1 (96.3-97.8)	85.6 (83.9-87.1)	72.3 (70.3-74.3)	43.8 (41.6-46)
Specificity % (95% CI)	65.4 (65.1-65.7)	91.3 (91.2-91.5)	95.4 (95.2-95.5)	98.8 (98.7-98.9)	35.3 (34.8-35.9)	76.0 (75.6-76.5)	86.1 (85.8-86.5)	96.1 (95.9-96.3)	22.4 (21.7-23.2)	63.4 (62.5-64.3)	77.1 (76.3-77.8)	92.6 (92.2-93.1)
PPV % (95% CI)	12.2 (11.8-12.5)	30.4 (29.5-31.3)	39.8 (38.6-41)	57.6 (55.6-59.6)	15.1 (14.6-15.5)	28.8 (28-29.7)	36.7 (35.6-37.8)	53.5 (51.6-55.3)	17.8 (17.1-18.6)	28.9 (27.7-30)	35.4 (33.9-36.9)	50.8 (48.5-53.2)
NPV % (95% CI)	99.1 (99-99.2)	98.3 (98.2-98.4)	97.6 (97.5-97.7)	96.3 (96.2-96.5)	98.5 (98.3-98.7)	97.1 (96.8-97.3)	95.6 (95.4-95.8)	92.8 (92.5-93)	97.8 (97.2-98.3)	96.2 (95.7-96.6)	94.1 (93.6-94.6)	90.5 (89.9-91)
LR+ (95% CI)	2.57 (2.53-2.6)	8.09 (7.86-8.33)	12.24 (11.76-12.74)	25.19 (23.36-27.15)	1.48 (1.46-1.49)	3.37 (3.29-3.46)	4.83 (4.66-5.00)	9.57 (8.95-10.24)	1.25 (1.24-1.27)	2.34 (2.27-2.41)	3.16 (3.02-3.29)	5.96 (5.49-6.46)
LR- (95% CI)	0.17 (0.16-0.19)	0.33 (0.31-0.34)	0.45 (0.44-0.47)	0.70 (0.69-0.72)	0.12 (0.11-0.14)	0.25 (0.24-0.27)	0.38 (0.37-0.40)	0.65 (0.63-0.67)	0.13 (0.1-0.17)	0.23 (0.2-0.25)	0.36 (0.33-0.39)	0.61 (0.58-0.63)
DOR (95% CI)	14.94 (13.62-16.42)	24.68 (23.06-26.41)	27.05 (25.3-28.92)	35.89 (32.85-39.24)	11.87 (10.2-13.91)	13.37 (12.3-14.55)	12.59 (11.7-13.56)	14.73 (13.52-16.05)	9.58 (7.44-12.6)	10.25 (9.02-11.7)	8.79 (7.91-9.79)	9.81 (8.77-10.98)

Abbreviations: DOR = diagnostic odds ratio, **FN** = false negatives, **FP** = false positives, **LR** = likelihood ratio, **N** = number, **NPV** = negative predictive value, **PPV** = positive predictive value, **TN** = true negatives, **TP** = true positives
