# Supplementary material for: NT-proBNP testing for heart failure diagnosis in people with atrial fibrillation: A diagnostic accuracy study
Source: PLoS Med. 2025 Oct 30;22(10):e1004550. doi: 10.1371/journal.pmed.1004550 (PMC12574882; doi:10.1371/journal.pmed.1004550)
Supplement: S2 Table — (PDF) [file pmed.1004550.s002.pdf]

Supplementary Table 2. Diagnostic test accuracy parameters for the diagnosis of HF using NT-proBNP level among **people with underweight (BMI <20kg/m2)** at NICE and ESC referral thresholds based on presence of pre-existing atrial fibrillation

	**With atrial fibrillation (n=637)**				**Without atrial fibrillation (n=4,905)**			
NT-proBNP threshold (pg/mL)	≥125	≥400	≥660	≥2000	≥125	≥400	≥660	≥2000
Prevalence % (95% CI)	28.4 (24.9-32.1)	28.4 (24.9-32.1)	28.4 (24.9-32.1)	28.4 (24.9-32.1)	11.2 (10.3-12.1)	11.2 (10.3-12.1)	11.2 (10.3-12.1)	11.2 (10.3-12.1)
TP, n	180	173	163	110	530	465	385	246
FN, n	1	8	18	71	18	83	163	302
FP, n	411	329	275	131	2977	1313	808	264
TN, n	45	127	181	325	1380	3044	3549	4093
Sensitivity % (95% CI)	99.4 (97-100)	95.6 (91.5-98.1)	90.1 (84.7-94.0)	60.8 (53.3-67.9)	96.7 (94.9-98.0)	84.9 (81.6-87.8)	70.3 (66.2-74.1)	44.9 (40.7-49.2)
Specificity % (95% CI)	9.9 (7.3-13.0)	27.9 (23.8-32.2)	39.7 (35.2-44.3)	71.3 (66.9-75.4)	31.7 (30.3-33.1)	69.9 (68.5-71.2)	81.5 (80.3-82.6)	93.9 (93.2-94.6)
PPV % (95% CI)	30.5 (26.8-34.3)	34.5 (30.3-38.8)	37.2 (32.7-41.9)	45.6 (39.2-52.2)	15.1 (13.9-16.3)	26.2 (24.1-28.3)	32.3 (29.6-35.0)	48.2 (43.8-52.7)
NPV % (95% CI)	97.8 (88.5-99.9)	94.1 (88.7-97.4)	91.0 (86.1-94.6)	82.1 (77.9-85.7)	98.7 (98.0-99.2)	97.3 (96.7-97.9)	95.6 (94.9-96.2)	93.1 (92.3-93.9)
LR+ (95% CI)	1.1 (1.07-1.14)	1.32 (1.24-1.41)	1.49 (1.37-1.63)	2.12 (1.76-2.55)	1.42 (1.38-1.45)	2.82 (2.66-2.98)	3.79 (3.49-4.12)	7.41 (6.38-8.6)
LR- (95% CI)	0.06 (0.01-0.4)	0.16 (0.08-0.32)	0.25 (0.16-0.39)	0.55 (0.45-0.67)	0.1 (0.07-0.16)	0.22 (0.18-0.26)	0.37 (0.32-0.42)	0.59 (0.54-0.63)
DOR (95% CI)	17.24 (3.77-406.01)	8.18 (4.15-18.66)	5.9 (3.59-10.28)	3.83 (2.68-5.52)	13.53 (8.68-22.58)	12.96 (10.23-16.63)	10.36 (8.51-12.67)	12.61 (10.23-15.56)

Abbreviations: DOR = diagnostic odds ratio**, FN** = false negatives**, FP** = false positives**, LR** = likelihood ratio**, N** = number**, NPV** = negative predictive value**, PPV** = positive predictive value**, TN** = true negatives**, TP** = true positives
