# Supplementary material for: NT-proBNP testing for heart failure diagnosis in people with atrial fibrillation: A diagnostic accuracy study
Source: PLoS Med. 2025 Oct 30;22(10):e1004550. doi: 10.1371/journal.pmed.1004550 (PMC12574882; doi:10.1371/journal.pmed.1004550)
Supplement: S3 Table — (PDF) [file pmed.1004550.s003.pdf]

Supplementary Table 3. Diagnostic test accuracy parameters for the diagnosis of HF using NT-proBNP level among **people with healthy weight (BMI 20 to 25kg/m2)** at NICE and ESC referral thresholds based on presence of pre-existing atrial fibrillation

	**With atrial fibrillation (n=3,972)**				**Without atrial fibrillation (n=26,949)**			
NT-proBNP threshold (pg/mL)	≥125	≥400	≥660	≥2000	≥125	≥400	≥660	≥2000
Prevalence % (95% CI)	27.1 (25.7-28.5)	27.1 (25.7-28.5)	27.1 (25.7-28.5)	27.1 (25.7-28.5)	8.9 (8.5-9.2)	8.9 (8.5-9.2)	8.9 (8.5-9.2)	8.9 (8.5-9.2)
TP, n	1066	1014	957	606	2302	2015	1711	1062
FN, n	10	62	119	470	87	374	678	1327
FP, n	2636	2040	1767	730	13669	5049	3006	927
TN, n	260	856	1129	2166	10891	19511	21554	23633
Sensitivity % (95% CI)	99.1 (98.3-99.6)	94.2 (92.7-95.6)	88.9 (86.9-90.8)	56.3 (53.3-59.3)	96.4 (95.5-97.1)	84.3 (82.8-85.8)	71.6 (69.8-73.4)	44.5 (42.4-46.5)
Specificity % (95% CI)	9.0 (8.0-10.1)	29.6 (27.9-31.3)	39.0 (37.2-40.8)	74.8 (73.2-76.4)	44.3 (43.7-45.0)	79.4 (78.9-79.9)	87.8 (87.3-88.2)	96.2 (96.0-96.5)
PPV % (95% CI)	28.8 (27.3-30.3)	33.2 (31.5-34.9)	35.1 (33.3-37.0)	45.4 (42.7-48.1)	14.4 (13.9-15.0)	28.5 (27.5-29.6)	36.3 (34.9-37.7)	53.4 (51.2-55.6)
NPV % (95% CI)	96.3 (93.3-98.2)	93.2 (91.4-94.8)	90.5 (88.7-92.0)	82.2 (80.7-83.6)	99.2 (99.0-99.4)	98.1 (97.9-98.3)	97.0 (96.7-97.2)	94.7 (94.4-95)
LR+ (95% CI)	1.09 (1.07-1.10)	1.34 (1.30-1.38)	1.46 (1.41-1.51)	2.23 (2.06-2.42)	1.73 (1.71-1.76)	4.1 (3.98-4.23)	5.85 (5.61-6.1)	11.78 (10.9-12.73)
LR- (95% CI)	0.1 (0.06-0.19)	0.19 (0.15-0.25)	0.28 (0.24-0.34)	0.58 (0.54-0.63)	0.08 (0.07-0.1)	0.2 (0.18-0.22)	0.32 (0.3-0.34)	0.58 (0.56-0.6)
DOR (95% CI)	10.35 (5.79-20.99)	6.84 (5.28-9.04)	5.13 (4.2-6.32)	3.82 (3.3-4.43)	21.04 (17.08-26.28)	20.81 (18.58-23.37)	18.09 (16.43-19.94)	20.4 (18.38-22.62)

Abbreviations: DOR = diagnostic odds ratio, **FN** = false negatives, **FP** = false positives, **LR** = likelihood ratio, **N** = number, **NPV** = negative predictive value, **PPV** = positive predictive value, **TN** = true negatives, **TP** = true positives
