# Supplementary material for: NT-proBNP testing for heart failure diagnosis in people with atrial fibrillation: A diagnostic accuracy study
Source: PLoS Med. 2025 Oct 30;22(10):e1004550. doi: 10.1371/journal.pmed.1004550 (PMC12574882; doi:10.1371/journal.pmed.1004550)
Supplement: S4 Table — (PDF) [file pmed.1004550.s004.pdf]

Supplementary Table 4. Diagnostic test accuracy parameters for the diagnosis of HF using NT-proBNP level among **people with overweight (BMI 25-30kg/m2)** at NICE and ESC referral thresholds based on presence of pre-existing atrial fibrillation

	**With atrial fibrillation (n=6,059)**				**Without atrial fibrillation (n=45,571)**			
NT-proBNP threshold (pg/mL)	≥125	≥400	≥660	≥2000	≥125	≥400	≥660	≥2000
Prevalence % (95% CI)	23.3 (22.3-24.4)	23.3 (22.3-24.4)	23.3 (22.3-24.4)	23.3 (22.3-24.4)	7.4 (7.2-7.7)	7.4 (7.2-7.7)	7.4 (7.2-7.7)	7.4 (7.2-7.7)
TP, n	1392	1317	1221	662	3145	2655	2190	1243
FN, n	21	96	192	751	231	721	1186	2133
FP, n	4072	3044	2504	803	19720	6355	3561	1033
TN, n	574	1602	2142	3843	22475	35840	38634	41162
Sensitivity % (95% CI)	98.5 (97.7-99.1)	93.2 (91.8-94.5)	86.4 (84.5-88.2)	46.9 (44.2-49.5)	93.2 (92.3-94.0)	78.6 (77.2-80.0)	64.9 (63.2-66.5)	36.8 (35.2-38.5)
Specificity % (95% CI)	12.4 (11.4-13.3)	34.5 (33.1-35.9)	46.1 (44.7-47.6)	82.7 (81.6-83.8)	53.3 (52.8-53.7)	84.9 (84.6-85.3)	91.6 (91.3-91.8)	97.6 (97.4-97.7)
PPV % (95% CI)	25.5 (24.3-26.7)	30.2 (28.8-31.6)	32.8 (31.3-34.3)	45.2 (42.6-47.8)	13.8 (13.3-14.2)	29.5 (28.5-30.4)	38.1 (36.8-39.3)	54.6 (52.5-56.7)
NPV % (95% CI)	96.5 (94.7-97.8)	94.3 (93.1-95.4)	91.8 (90.6-92.9)	83.7 (82.6-84.7)	99.0 (98.8-99.1)	98.0 (97.9-98.2)	97.0 (96.8-97.2)	95.1 (94.9-95.3)
LR+ (95% CI)	1.12 (1.11-1.14)	1.42 (1.39-1.46)	1.6 (1.55-1.66)	2.71 (2.49-2.95)	1.99 (1.97-2.02)	5.22 (5.07-5.37)	7.69 (7.38-8.00)	15.04 (13.96-16.21)
LR- (95% CI)	0.12 (0.08-0.19)	0.2 (0.16-0.24)	0.29 (0.26-0.34)	0.64 (0.61-0.68)	0.13 (0.11-0.15)	0.25 (0.24-0.27)	0.38 (0.37-0.4)	0.65 (0.63-0.66)
DOR (95% CI)	9.27 (6.13-14.86)	7.21 (5.84-9)	5.44 (4.63-6.41)	4.22 (3.71-4.8)	15.51 (13.58-17.79)	20.76 (19.05-22.64)	20.03 (18.52-21.69)	23.22 (21.15-25.49)

Abbreviations: DOR = diagnostic odds ratio**, FN** = false negatives**, FP** = false positives**, LR** = likelihood ratio**, N** = number**, NPV** = negative predictive value**, PPV** = positive predictive value**, TN** = true negatives**, TP** = true positives
