# Supplementary material for: NT-proBNP testing for heart failure diagnosis in people with atrial fibrillation: A diagnostic accuracy study
Source: PLoS Med. 2025 Oct 30;22(10):e1004550. doi: 10.1371/journal.pmed.1004550 (PMC12574882; doi:10.1371/journal.pmed.1004550)
Supplement: S5 Table — (PDF) [file pmed.1004550.s005.pdf]

Supplementary Table 5. Diagnostic test accuracy parameters for the diagnosis of HF using NT-proBNP level among **people with obesity stage 1 (BMI 30-35kg/m2)** at NICE and ESC referral thresholds based on presence of pre-existing atrial fibrillation

	**With atrial fibrillation (n=3,716)**				**Without atrial fibrillation (n=31,988)**			
NT-proBNP threshold (pg/mL)	≥125	≥400	≥660	≥2000	≥125	≥400	≥660	≥2000
Prevalence % (95% CI)	22.5 (21.1-23.8)	22.5 (21.1-23.8)	22.5 (21.1-23.8)	22.5 (21.1-23.8)	6.5 (6.3-6.8)	6.5 (6.3-6.8)	6.5 (6.3-6.8)	6.5 (6.3-6.8)
TP, n	830	773	691	328	1892	1495	1224	627
FN, n	5	62	144	507	195	592	863	1460
FP, n	2410	1726	1334	329	12186	3474	1918	469
TN, n	471	1155	1547	2552	17715	26427	27983	29432
Sensitivity % (95% CI)	99.4 (98.6-99.8)	92.6 (90.6-94.3)	82.8 (80.0-85.3)	39.3 (36.0-42.7)	90.7 (89.3-91.9)	71.6 (69.6-73.6)	58.6 (56.5-60.8)	30.0 (28.1-32.1)
Specificity % (95% CI)	16.3 (15.0-17.8)	40.1 (38.3-41.9)	53.7 (51.9-55.5)	88.6 (87.4-89.7)	59.2 (58.7-59.8)	88.4 (88.0-88.7)	93.6 (93.3-93.9)	98.4 (98.3-98.6)
PPV % (95% CI)	25.6 (24.1-27.2)	30.9 (29.1-32.8)	34.1 (32.1-36.2)	49.9 (46.0-53.8)	13.4 (12.9-14.0)	30.1 (28.8-31.4)	39.0 (37.2-40.7)	57.2 (54.2-60.2)
NPV % (95% CI)	98.9 (97.6-99.7)	94.9 (93.5-96.1)	91.5 (90.1-92.8)	83.4 (82.1-84.7)	98.9 (98.7-99.1)	97.8 (97.6-98.0)	97.0 (96.8-97.2)	95.3 (95.0-95.5)
LR+ (95% CI)	1.19 (1.17-1.21)	1.55 (1.49-1.6)	1.79 (1.70-1.88)	3.44 (3.01-3.93)	2.22 (2.18-2.27)	6.17 (5.92-6.43)	9.14 (8.64-9.67)	19.15 (17.14-21.41)
LR- (95% CI)	0.04 (0.02-0.09)	0.19 (0.15-0.24)	0.32 (0.28-0.37)	0.69 (0.65-0.73)	0.16 (0.14-0.18)	0.32 (0.3-0.34)	0.44 (0.42-0.47)	0.71 (0.69-0.73)
DOR (95% CI)	31.43 (14.48-89.14)	8.32 (6.41-10.99)	5.56 (4.59-6.77)	5.02 (4.19-6.01)	14.09 (12.17-16.41)	19.2 (17.36-21.26)	20.69 (18.75-22.84)	26.94 (23.65-30.73)

Abbreviations: DOR = diagnostic odds ratio**, FN** = false negatives**, FP** = false positives**, LR** = likelihood ratio**, N** = number**, NPV** = negative predictive value**, PPV** = positive predictive value**, TN** = true negatives**, TP** = true positives
