# Supplementary material for: NT-proBNP testing for heart failure diagnosis in people with atrial fibrillation: A diagnostic accuracy study
Source: PLoS Med. 2025 Oct 30;22(10):e1004550. doi: 10.1371/journal.pmed.1004550 (PMC12574882; doi:10.1371/journal.pmed.1004550)
Supplement: S6 Table — (PDF) [file pmed.1004550.s006.pdf]

Supplementary Table 6. Diagnostic test accuracy parameters for the diagnosis of HF using NT-proBNP level among **people with obesity (BMI 35-40kg/m2)** at NICE and ESC referral thresholds based on presence of pre-existing atrial fibrillation

	**Withatrial fibrillation (n=1,523)**				**Without atrial fibrillation (n=14,443)**			
NT-proBNP threshold (pg/mL)	≥125	≥400	≥660	≥2000	≥125	≥400	≥660	≥2000
Prevalence % (95% CI)	22.4 (20.3-24.6)	22.4 (20.3-24.6)	22.4 (20.3-24.6)	22.4 (20.3-24.6)	6.3 (5.9-6.7)	6.3 (5.9-6.7)	6.3 (5.9-6.7)	6.3 (5.9-6.7)
TP, n	334	311	282	109	799	601	460	181
FN, n	7	30	59	232	104	302	443	722
FP, n	988	691	523	117	4978	1309	719	167
TN, n	194	491	659	1065	8562	12231	12821	13373
Sensitivity % (95% CI)	97.9 (95.8-99.2)	91.2 (87.7-94)	82.7 (78.3-86.6)	32.0 (27.0-37.2)	88.5 (86.2-90.5)	66.6 (63.4-69.6)	50.9 (47.6-54.2)	20.0 (17.5-22.8)
Specificity % (95% CI)	16.4 (14.3-18.6)	41.5 (38.7-44.4)	55.8 (52.9-58.6)	90.1 (88.3-91.7)	63.2 (62.4-64.0)	90.3 (89.8-90.8)	94.7 (94.3-95.1)	98.8 (98.6-98.9)
PPV % (95% CI)	25.3 (22.9-27.7)	31.0 (28.2-34)	35.0 (31.7-38.4)	48.2 (41.6-55.0)	13.8 (13.0-14.7)	31.5 (29.4-33.6)	39.0 (36.2-41.9)	52.0 (46.6-57.4)
NPV % (95% CI)	96.5 (93.0-98.6)	94.2 (91.9-96.1)	91.8 (89.5-93.7)	82.1 (79.9-84.2)	98.8 (98.5-99.0)	97.6 (97.3-97.9)	96.7 (96.3-97.0)	94.9 (94.5-95.2)
LR+ (95% CI)	1.17 (1.14-1.21)	1.56 (1.47-1.65)	1.87 (1.72-2.03)	3.23 (2.56-4.07)	2.41 (2.33-2.49)	6.88 (6.42-7.38)	9.59 (8.72-10.56)	16.25 (13.32-19.83)
LR- (95% CI)	0.13 (0.06-0.26)	0.21 (0.15-0.3)	0.31 (0.24-0.39)	0.76 (0.7-0.81)	0.18 (0.15-0.22)	0.37 (0.34-0.41)	0.52 (0.48-0.55)	0.81 (0.78-0.84)
DOR (95% CI)	9.16 (4.6-21.86)	7.33 (5.03-11.06)	6 (4.46-8.2)	4.27 (3.17-5.75)	13.19 (10.78-16.32)	18.58 (16.02-21.61)	18.51 (15.92-21.51)	20.06 (16.05-25.09)

Abbreviations: DOR = diagnostic odds ratio**, FN** = false negatives**, FP** = false positives**, LR** = likelihood ratio**, N** = number**, NPV** = negative predictive value**, PPV** = positive predictive value**, TN** = true negatives**, TP** = true positives
