# Supplementary material for: NT-proBNP testing for heart failure diagnosis in people with atrial fibrillation: A diagnostic accuracy study
Source: PLoS Med. 2025 Oct 30;22(10):e1004550. doi: 10.1371/journal.pmed.1004550 (PMC12574882; doi:10.1371/journal.pmed.1004550)
Supplement: S7 Table — (PDF) [file pmed.1004550.s007.pdf]

**Supplementary Table 7.** Diagnostic test accuracy parameters for the diagnosis of HF using NT-proBNP level among **people with obesity stage 3 (BMI ≥40kg/m2)** at NICE and ESC referral thresholds based on presence of pre-existing atrial fibrillation

| | **With atrial fibrillation (n=861)** | | | | **Without atrial fibrillation (n=8,982)** | | | |
|---|---|---|---|---|---|---|---|---|
| NT-proBNP threshold (pg/mL) | ≥125 | ≥400 | ≥660 | ≥2000 | ≥125 | ≥400 | ≥660 | ≥2000 |
| Prevalence % (95% CI) | 19.6 (17.0-22.4) | 19.6 (17.0-22.4) | 19.6 (17.0-22.4) | 19.6 (17.0-22.4) | 6.6 (6.1-7.1) | 6.6 (6.1-7.1) | 6.6 (6.1-7.1) | 6.6 (6.1-7.1) |
| TP, n | 166 | 150 | 131 | 50 | 513 | 359 | 285 | 133 |
| FN, n | 3 | 19 | 38 | 119 | 79 | 233 | 307 | 459 |
| FP, n | 553 | 380 | 273 | 37 | 2978 | 768 | 418 | 86 |
| TN, n | 139 | 312 | 419 | 655 | 5412 | 7622 | 7972 | 8304 |
| Sensitivity % (95% CI) | 98.2 (94.9-99.6) | 88.8 (83.0-93.1) | 77.5 (70.5-83.6) | 29.6 (22.8-37.1) | 86.7 (83.6-89.3) | 60.6 (56.6-64.6) | 48.1 (44.1-52.3) | 22.5 (19.2-26.0) |
| Specificity % (95% CI) | 20.1 (17.2-23.3) | 45.1 (41.3-48.9) | 60.5 (56.8-64.2) | 94.7 (92.7-96.2) | 64.5 (63.5-65.5) | 90.8 (90.2-91.5) | 95.0 (94.5-95.5) | 99.0 (98.7-99.2) |
| PPV % (95% CI) | 23.1 (20.1-26.3) | 28.3 (24.5-32.3) | 32.4 (27.9-37.2) | 57.5 (46.4-68.0) | 14.7 (13.5-15.9) | 31.9 (29.1-34.7) | 40.5 (36.9-44.3) | 60.7 (53.9-67.2) |
| NPV % (95% CI) | 97.9 (94.0-99.6) | 94.3 (91.2-96.5) | 91.7 (88.8-94) | 84.6 (81.9-87.1) | 98.6 (98.2-98.9) | 97.0 (96.6-97.4) | 96.3 (95.9-96.7) | 94.8 (94.3-95.2) |
| LR+ (95% CI) | 1.23 (1.18-1.28) | 1.62 (1.48-1.76) | 1.96 (1.74-2.22) | 5.53 (3.75-8.18) | 2.44 (2.34-2.55) | 6.62 (6.03-7.27) | 9.66 (8.52-10.95) | 21.92 (16.93-28.37) |
| LR- (95% CI) | 0.09 (0.03-0.27) | 0.25 (0.16-0.38) | 0.37 (0.28-0.49) | 0.74 (0.67-0.82) | 0.21 (0.17-0.25) | 0.43 (0.39-0.48) | 0.55 (0.5-0.59) | 0.78 (0.75-0.82) |
| DOR (95% CI) | 13.22 (4.92-55.8) | 6.43 (3.99-10.94) | 5.27 (3.59-7.89) | 7.4 (4.65-11.9) | 11.78 (9.31-15.09) | 15.28 (12.76-18.33) | 17.69 (14.65-21.39) | 27.93 (21-37.34) |

**Abbreviations: DOR** = diagnostic odds ratio, **FN** = false negatives, **FP** = false positives, **LR** = likelihood ratio, **N** = number, **NPV** = negative predictive value, **PPV** = positive predictive value, **TN** = true negatives, **TP** = true positives
