# Supplementary material for: NT-proBNP testing for heart failure diagnosis in people with atrial fibrillation: A diagnostic accuracy study
Source: PLoS Med. 2025 Oct 30;22(10):e1004550. doi: 10.1371/journal.pmed.1004550 (PMC12574882; doi:10.1371/journal.pmed.1004550)
Supplement: S8 Table — (PDF) [file pmed.1004550.s008.pdf]

Supplementary Table 8. Diagnostic test accuracy parameters for the diagnosis of HF using NT-proBNP level **by sex** at NICE and ESC referral thresholds for those **without** pre-existing atrial fibrillation

	**Males (56,923)**				**Females (n=81,021)**			
NT-proBNP threshold (pg/mL)	≥125	≥400	≥660	≥2000	≥125	≥400	≥660	≥2000
Prevalence % (95% CI)	9.1 (8.89.3)	9.1 (8.89.3	9.1 (8.89.3	9.1 (8.89.3	6.5 (6.36.7)	6.5 (6.3-6.7)	6.5 (6.36.7)	6.5 (6.36.7)
TP, n	4762	4040	3459	2025	4919	3989	3170	1705
FN, n	390	1112	1693	3127	346	1276	2095	3560
FP, n	21519	7911	4891	1513	37352	11337	6148	1633
TN, n	30252	43860	46880	50258	38404	64419	69608	74123
Sensitivity % (95% CI)	92.4 (91.7-93.1)	78.4 (77.3-79.5)	67.1 (65.8-68.4)	39.3 (38-40.7)	93.4 (92.7-94.1)	75.8 (74.6-76.9)	60.2 (58.9-61.5)	32.4 (31.1-33.7)
Specificity % (95% CI)	58.4 (58-58.9)	84.7 (84.4-85)	90.6 (90.3-90.8)	97.1 (96.9-97.2)	50.7 (50.3-51.1)	85 (84.8-85.3)	91.9 (91.7-92.1)	97.8 (97.7-97.9)
PPV % (95% CI)	18.1 (17.7-18.6)	33.8 (33-34.7)	41.4 (40.4-42.5)	57.2 (55.6-58.9)	11.6 (11.3-11.9)	26 (25.3-26.7)	34 (33.1-35)	51.1 (49.4-52.8)
NPV % (95% CI)	98.7 (98.6-98.8)	97.5 (97.4-97.7)	96.5 (96.3-96.7)	94.1 (93.9-94.3)	99.1 (99-99.2)	98.1 (97.9-98.2)	97.1 (97-97.2)	95.4 (95.3-95.6)
LR+ (95% CI)	2.22 (2.2-2.25)	5.13 (5.01-5.26)	7.11 (6.88-7.34)	13.45 (12.66-14.28)	1.89 (1.88-1.91)	5.06 (4.95-5.18)	7.42 (7.18-7.66)	15.02 (14.12-15.98)
LR- (95% CI)	0.13 (0.12-0.14)	0.25 (0.24-0.27)	0.36 (0.35-0.38)	0.63 (0.61-0.64)	0.13 (0.12-0.14)	0.29 (0.27-0.3)	0.43 (0.42-0.45)	0.69 (0.68-0.7)
DOR (95% CI)	17.16 (15.47-19.07)	20.14 (18.77-21.61)	19.58 (18.35-20.9)	21.51 (19.94-23.21)	14.61 (13.11-16.33)	17.76 (16.63-18.97)	17.13 (16.12-18.21)	21.74 (20.15-23.45)

Abbreviations: DOR = diagnostic odds ratio**, FN** = false negatives, **FP** = false positives, **LR** = likelihood ratio, **N** = number, **NPV** = negative predictive value, **PPV** = positive predictive value, **TN** = true negatives, **TP** = true positives
