# Supplementary material for: NT-proBNP testing for heart failure diagnosis in people with atrial fibrillation: A diagnostic accuracy study
Source: PLoS Med. 2025 Oct 30;22(10):e1004550. doi: 10.1371/journal.pmed.1004550 (PMC12574882; doi:10.1371/journal.pmed.1004550)
Supplement: S9 Table — (PDF) [file pmed.1004550.s009.pdf]

Supplementary Table 9. Diagnostic test accuracy parameters for the diagnosis of HF using NT-proBNP level **by sex** at NICE and ESC referral thresholds for those with pre-existing atrial fibrillation

	**Males** (n=8,960)				**Females** (n=8,443)			
**NT-proBNP threshold (pg/mL)**	**≥125**	**≥400**	**≥660**	**≥2000**	**≥125**	**≥400**	**≥660**	**≥2000**
**Prevalence % (95% CI)**	25.1 (24.2-26.0)	25.1 (24.2-26.0)	25.1 (24.2-26.0)	25.1 (24.2-26.0)	22.7 (21.8-23.6)	22.7 (21.8-23.6)	22.7 (21.8-23.6)	22.7 (21.8-23.6)
**TP, n**	2,224	2,094	1,911	1,017	1,896	1,790	1,671	927
**FN, n**	26	156	339	1,233	22	128	247	991
**FP, n**	5,707	4,314	3,480	1,053	5,787	4,226	3,479	1,225
**TN, n**	1,003	2,396	3,230	5,657	738	2,299	3,046	5,300
**Sensitivity % (95% CI)**	98.8 (98.3-99.2)	93.1 (91.9-94.1)	84.9 (83.4-86.4)	45.2 (43.1-47.3)	98.9 (98.3-99.3)	93.3 (92.1-94.4)	87.1 (85.5-88.6)	48.3 (46.1-50.6)
**Specificity % (95% CI)**	14.9 (14.1-15.8)	35.7 (34.6-36.9)	48.1 (46.9-49.3)	84.3 (83.4-85.2)	11.3 (10.6-12.1)	35.2 (34.1-36.4)	46.7 (45.5-47.9)	81.2 (80.3-82.2)
**PPV % (95% CI)**	28.0 (27.1-29.0)	32.7 (31.5-33.8)	35.4 (34.2-36.7)	49.1 (47.0-51.3)	24.7 (23.7-25.7)	29.8 (28.6-30.9)	32.4 (31.2-33.7)	43.1 (41-45.2)
**NPV % (95% CI)**	97.5 (96.3-98.3)	93.9 (92.9-94.8)	90.5 (89.5-91.4)	82.1 (81.2-83.0)	97.1 (95.7-98.2)	94.7 (93.8-95.6)	92.5 (91.5-93.4)	84.2 (83.3-85.1)
**LR+ (95% CI)**	1.16 (1.15-1.17)	1.45 (1.42-1.48)	1.64 (1.59-1.69)	2.88 (2.68-3.09)	1.11 (1.1-1.13)	1.44 (1.41-1.47)	1.63 (1.59-1.68)	2.57 (2.4-2.76)
**LR- (95% CI)**	0.08 (0.05-0.11)	0.19 (0.17-0.23)	0.31 (0.28-0.35)	0.65 (0.63-0.68)	0.1 (0.07-0.15)	0.19 (0.16-0.22)	0.28 (0.24-0.31)	0.64 (0.61-0.67)
**DOR (95% CI)**	14.94 (10.31-22.71)	7.45 (6.30-8.86)	5.23 (4.62-5.93)	4.43 (3.99-4.93)	10.91 (7.3-17.26)	7.6 (6.33-9.2)	5.92 (5.14-6.84)	4.05 (3.63-4.51)

Abbreviations: DOR = diagnostic odds ratio, **FN** = false negatives, **FP** = false positives, **LR** = likelihood ratio, **N** = number, **NPV** = negative predictive value, **PPV** = positive predictive value, **TN** = true negatives, **TP** = true positives
