# Supplementary material for: NT-proBNP testing for heart failure diagnosis in people with atrial fibrillation: A diagnostic accuracy study
Source: PLoS Med. 2025 Oct 30;22(10):e1004550. doi: 10.1371/journal.pmed.1004550 (PMC12574882; doi:10.1371/journal.pmed.1004550)
Supplement: S10 Table — (PDF) [file pmed.1004550.s010.pdf]

Supplementary Table 10. Proportion of patients undergoing NT-proBNP testing who would be referred for further assessment and have a diagnosis of heart failure confirmed, comparing between different NT-proBNP thresholds and based on presence of pre-existing atrial fibrillation

	With AF (n=17,403)				Without AF (n=137,944)			
NT-proBNP threshold (pg/mL)	≥125	≥400	≥660	≥2000	≥125	≥400	≥660	≥2000
Total patients referred (% of those tested)	15,614 (89.7%)	12,424 (71.4%)	10,541 (60.6%)	4,222 (24.3%)	68,552 (49.7%)	27,277 (19.8%)	17,668 (12.8%)	6,876 (4.98%)
Reduction in referrals compared-125pg/ml (% of those tested)	N/A	3,190 (18.3%)	5,073 (29.2%)	11,392 (65.5%)	N/A	41,275 (29.9%)	50,884 (36.9%)	61,676 (44.7%)
Total referrals that are diagnosed with heart failure (% of those referred)	4,120 (26.4%)	3,884 (31.3%)	3,582 (34.0%)	1,944 (46.0%)	9,681 (14.1%)	8,029 (29.4%)	6,629 (37.5%)	3,730 (54.2%)
Total patients with missed or delayed diagnosis of heart failure (% of those tested)	48 (0.28%)	284 (1.63%)	586 (3.37%)	2,224 (12.8%)	736 (0.53%)	2,388 (1.73%)	3,788 (2.75%)	6,687 (4.85%)
Additional missed or delayed diagnoses made compared-125pg/ml (% of avoided referrals)	N/A	236 (7.40%)	538 (10.6%)	2,176 (19.1%)	N/A	1,652 (4.00%)	3,052 (6.00%)	5,951 (9.65%)
