# Supplementary material for: NT-proBNP testing for heart failure diagnosis in people with atrial fibrillation: A diagnostic accuracy study
Source: PLoS Med. 2025 Oct 30;22(10):e1004550. doi: 10.1371/journal.pmed.1004550 (PMC12574882; doi:10.1371/journal.pmed.1004550)
Supplement: S1 Appendix — (PDF) [file pmed.1004550.s011.pdf]

Appendix 1. Codes used to identify people undergoing NT-proBNP testing and diagnosed with heart failure or atrial fibrillation within the study

**Codes for N-terminal pro B-type natriuretic peptide**		
medcode	readcode	readterm
108309	44AV.00	Serum N-terminal pro B-type natriuretic peptide conc
27097	44AP.00	Serum pro-brain natriuretic peptide level
68734	4Q2B.00	N terminal pro-brain natriuretic peptide level
108253	44AW.00	Plasma N-terminal pro B-type natriuretic peptide conc
14140	44AN.00	Plasma pro-brain natriuretic peptide level
**Codes for heart failure**		
medcode	readcode	readterm
398	G580.00	Congestive heart failure
884	G581.00	Left ventricular failure
2062	G58..00	Heart failure
2906	G580.11	Congestive cardiac failure
12627	9N0k.00	Seen in heart failure clinic
4024	G58z.00	Heart failure NOS
19002	9N2p.00	Seen by community heart failure nurse
17851	8HBE.00	Heart failure follow-up
12366	662T.00	Congestive heart failure monitoring
30779	662W.00	Heart failure annual review
1223	G58..11	Cardiac failure
9913	1O1..00	Heart failure confirmed
13189	662g.00	New York Heart Association classification - class II
5942	G581.13	Impaired left ventricular function
72965	9Or3.00	Heart failure monitoring first letter
18853	662f.00	New York Heart Association classification - class I
19066	662h.00	New York Heart Association classification - class III
32911	9Or..00	Heart failure monitoring administration
19380	9Or0.00	Heart failure review completed
46672	388D.00	New York Heart Assoc classification heart failure symptoms
15058	14A6.00	H/O: heart failure
70619	8HHz.00	Referral to heart failure exercise programme

5255	G581000	Acute left ventricular failure
83502	662p.00	Heart failure 6 month review
32671	G580100	Chronic congestive heart failure
32945	8CL3.00	Heart failure care plan discussed with patient
9524	G580.14	Biventricular failure
10079	G580.12	Right heart failure
72386	9Or4.00	Heart failure monitoring second letter
60099	67D4.00	Heart failure information given to patient
27884	G580200	Decompensated cardiac failure
26115	8HHb.00	Referral to heart failure nurse
103732	8CMK.00	Has heart failure management plan
17278	G58z.12	Cardiac failure NOS
23707	G580000	Acute congestive heart failure
95835	679X.00	Heart failure education
27964	G582.00	Acute heart failure
10154	G580.13	Right ventricular failure
48897	8HTL.00	Referral to heart failure clinic
26242	ZRad.00	New York Heart Assoc classification heart failure symptoms
23481	G581.11	Asthma - cardiac
110101	8I98.00	Heart failure rehabilitation programme not available
51214	662i.00	New York Heart Association classification - class IV
89650	9Or5.00	Heart failure monitoring third letter
106894	8IE1.00	Referral to heart failure exercise programme declined
101138	G583.00	Heart failure with normal ejection fraction
43618	G581.12	Pulmonary oedema - acute
32898	8H2S.00	Admit heart failure emergency
90193	9Or1.00	Heart failure monitoring telephone invite
83481	9N4w.00	Did not attend heart failure clinic
11424	G580300	Compensated cardiac failure
104275	G584.00	Right ventricular failure
106897	G583.12	Heart failure with preserved ejection fraction
22262	G1yz100	Rheumatic left ventricular failure
90192	9Or2.00	Heart failure monitoring verbal invite
12590	G58z.11	Weak heart

46912	14AM.00	H/O: Heart failure in last year
106680	8HTL000	Referral to rapid access heart failure clinic
102585	8HgD.00	Discharge from heart failure nurse service
106198	661M500	Heart failure self-management plan agreed
94870	G580400	Congestive heart failure due to valvular disease
106008	8CMW800	Heart failure clinical pathway
101137	G583.11	HFNEF - heart failure with normal ejection fraction
95021	9N4s.00	Did not attend practice nurse heart failure clinic
21837	G232.00	Hypertensive heart&renal dis wth (congestive) heart failure
105002	679W100	Education about deteriorating heart failure
69062	9N6T.00	Referred by heart failure nurse specialist
71235	8Hk0.00	Referred to heart failure education group
105542	8CeC.00	Preferred place of care for next exacerbation heart failure
107981	8IE0.00	Referral to heart failure education group declined
91288	8Hg8.00	Discharge from practice nurse heart failure clinic
66306	SP11111	Heart failure as a complication of care
111428	2JZ..00	On optimal heart failure therapy
96799	G5y4z00	Post cardiac operation heart failure NOS
**Codes for atrial fibrillation**		
medcode	READcode	Description
1268	G573200	Paroxysmal atrial fibrillation
96277	G573400	Permanent atrial fibrillation
35127	G573300	Non-rheumatic atrial fibrillation
1664	G573000	Atrial fibrillation
96076	G573500	Persistent atrial fibrillation
2212	G573.00	Atrial fibrillation and flutter
23437	G573z00	Atrial fibrillation and flutter NOS
3757	3272	ECG: atrial fibrillation
