# Supplementary material for: NT-proBNP testing for heart failure diagnosis in people with atrial fibrillation: A diagnostic accuracy study
Source: PLoS Med. 2025 Oct 30;22(10):e1004550. doi: 10.1371/journal.pmed.1004550 (PMC12574882; doi:10.1371/journal.pmed.1004550)

Supplementary Figure 2. ROC curve of NT-proBNP among people **without pre-existing atrial fibrillation**, comparing between categories of body mass index

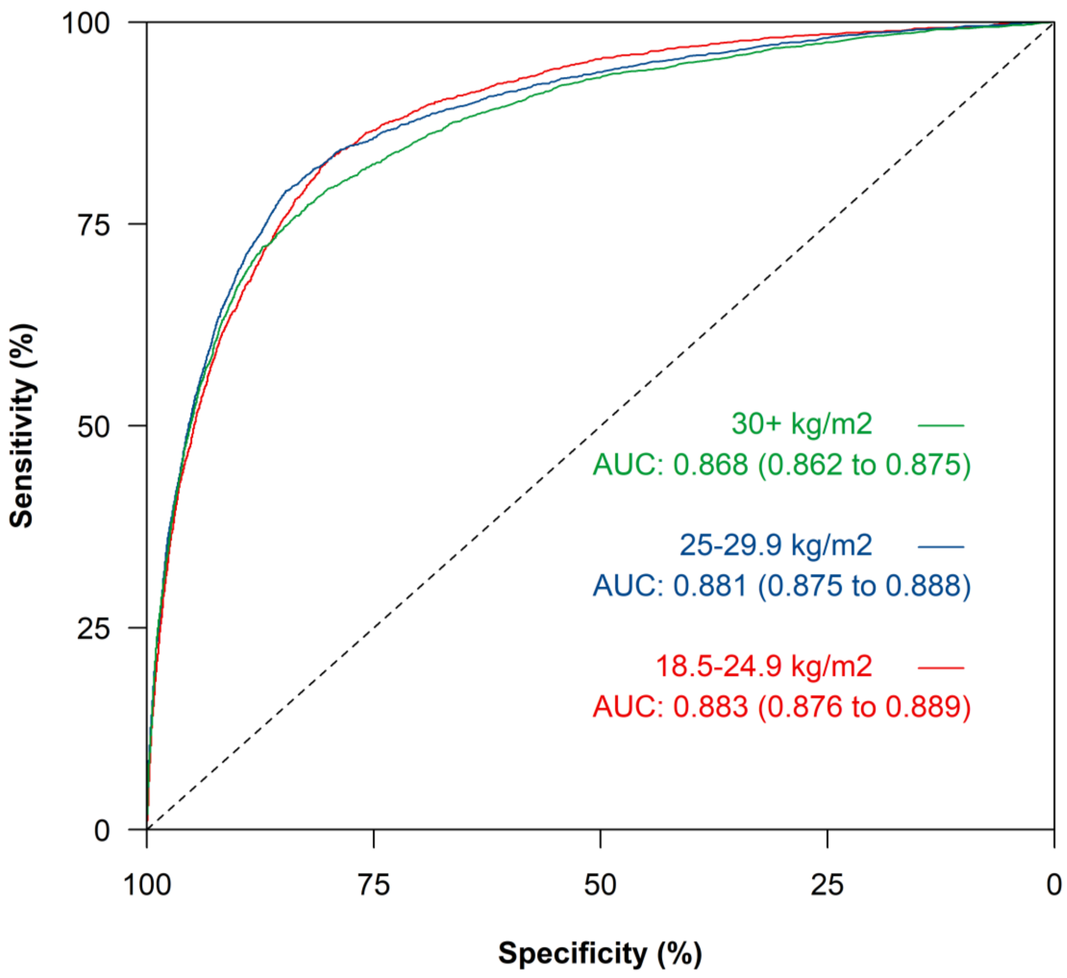

Supplement: S2 Fig — (PDF) [file pmed.1004550.s013.pdf]
