# Supplementary material for: NT-proBNP testing for heart failure diagnosis in people with atrial fibrillation: A diagnostic accuracy study
Source: PLoS Med. 2025 Oct 30;22(10):e1004550. doi: 10.1371/journal.pmed.1004550 (PMC12574882; doi:10.1371/journal.pmed.1004550)
Supplement: Attachment — Submitted filename: DiagnoseNP in AF PLoS reviewer response 10.7.25.docx [file pmed.1004550.s017.docx]

Comments from the academic editor:

The topic is interesting and providing more precise cutoffs for the diagnosis of HF is valuable, considering population heterogeneity where factors such as AF and age play significant roles. The use of a large-scale, real-world UK database further strengthens the reliability of the data.

However, the key issue is that BNP/NT-proBNP is primarily used as a rule-out biomarker for HF diagnosis. The current manuscript does not adequately address this perspective, focusing instead on general predictive performance as measured by AUC. Additionally, there are important questions raised by all other reviewers regarding the criteria used for population selection, particularly in how AF and HF are defined within the database.

Thank you for arranging for our paper to receive such comprehensive peer-review. We are very grateful for the time each reviewer has taken to comment on our work, and we have considered their feedback carefully. In revising our paper we have attempted to address all of the issues raised, including providing greater clarity around the purpose of NT-proBNP in primary care as a rule-out test for heart failure in people with and without atrial fibrillation (AF). More details are provided in the point-by-point response below.

We have also carefully checked the PLOS Medicine formatting requirements and made the necessary changes, such as including an Author Summary, updating the referencing format and moving Figure captions to their positon within the text.

-----------------------------------------------------------
Comments from the reviewers:

Reviewer #1: This article is on the effectiveness of NtproBNP as a diagnostic marker of heart failure in those with and without heart failure. Other reviewers will be better placed to comment on the clinical aspects of the study and its potential impact. However, in my opinion the statistical methods are appropriate given the available data, and the conclusions taken from the analysis are reasonable.

Thank you for reviewing the article and for these positive comments.

As a result I only have minor comments on the article:

1. In the tables it is being shown that there are thousands of individuals (2588 with AF and 9782 without) with HF diagnosed 6 or more weeks after the NtproBNP test that are nonetheless being considered as 'without heart failure'. As far as I can tell the rationale for this time cut off is not discussed within the report and also the implications that a reasonably high proportion (~5-10%) of this population do go on to develop heart failure. If known, it potentially also would be useful to report on more exact measures of time (months, years) the status is confirmed as opposed to just reporting it more than six weeks.

We report on the time between the NP test and heart failure diagnosis at these time points because they reflect the NICE guideline recommendations that people with an NT-proBNP ≥2,000pg/mL should be referred to a specialist for assessment of suspected heart failure within two weeks, whilst those with a NT-proBNP of 400pg/mL or more should be seen within six weeks. In practice, many patients wait longer than for this for a specialist assessment hence including diagnoses made within six months of the index test date. We have added an explanation for these timeframes to the Methods (lines 157-160):

*“NICE recommend people with an NT-proBNP ≥2000pg/mL are seen within two weeks of referral and people with an NT-proBNP ≥400pg/mL are seen within six weeks so we report on the proportion of HF diagnoses made within those timeframes.”*

We apologise that there was an error in Table 1, reporting that some people with and without atrial fibrillation were recorded as having a time between NTproBNP test and heart failure diagnosis of more than six weeks, but also included in the column ‘Not diagnosed with heart failure’. This was an error and it has been corrected; nobody in the ‘Not diagnosed with heart failure’ column was diagnosed with heart failure. Thank you for bringing this to our attention.

2. Although the sensitivity, specificity and AUC using previously reported thresholds are reported, I don't think there is any proposal as to what in theory the best thresholds could be for this AF population using the available data under different sensitivity/specificity considerations. To some extent this can be read from figure 1, but could be useful to have exact values reported for different scenarios, eg if there is a need for >99% sensitivity for a screening tool what threshold and specificity would result.

It is challenging to suggest a single optimal threshold across healthcare settings, particularly in people with AF, where NT-proBNP performs relatively poorly. A relatively low threshold would offer high sensitivity and negative predictive value so that very few cases of heart failure would be missed, but this approach would have significant resource implications because almost all patients with AF would need to be referred for an echocardiogram and further assessment. In the NHS there are already extended waiting times for specialist heart failure services and lowering the threshold to 125pg/ml in line with European guidance would add considerable strain to this system. Therefore, the UK might consider maintaining a threshold of 400pg/mL or increasing this to 660pg/mL among people with AF to prioritise those most likely to have heart failure for further testing. In contrast, healthcare systems with good access to echocardiography and shorter waits for heart failure specialist services may prefer setting a relatively low threshold in line with European guidance to avoid missed cases.

We do recognise the clinical importance of clear referral thresholds and so have adjusted the Discussion text (lines 361 to 363) to suggest a single proposed threshold in people with AF:

*“We suggest a higher threshold of 660pg/mL is considered to account for the fact that AF is associated with raised NT-proBNP levels to help target echocardiogram resources at those most likely to have HF.”*

Later in the Discussion (line 371) we make a recommendation regarding people without AF:

*“The ESC threshold of 125pg/mL may therefore be most appropriate for people without AF.”*

We seek to summarise the importance of considering the context of a healthcare system when establishing referral thresholds in the final paragraph before the Conclusion (lines 372 to 374) as follows:

*“Ultimately the threshold may be determined by healthcare system factors, such as access to echocardiography, where higher NP levels could support a fast-track referral. Implementation studies could help determine the clinical and cost impact of changing the NT-proBNP threshold.”*

3. Other than in the conclusions, there is no description as to why the high sensitivity/low specificity is appropriate for this context in using NtProBNP as an initial screening tool.

Thank you for this suggestion. We agree that making clear the context in which NT-proBNP is being used and it’s purpose as a ‘rule-out’ test are important to establish early in the paper so we have added significant detail regarding testing and the implications of a positive or negative result as follows (Methods, lines 128 to 137):

*“NT-proBNP is recommended to assess for heart failure among people with symptoms such as breathlessness, peripheral oedema or fatigue. NT-proBNP is used as a ‘rule out’ test in primary care, because patients without an elevated result are generally not referred on for further assessment of heart failure. Symptomatic patients with an elevated NT-proBNP should be referred for a specialist assessment to confirm or refute the diagnosis of heart failure, including further tests such as an echocardiogram. NT-proBNP thresholds therefore need to be set at a level that prioritises high sensitivity and negative predictive value (NPV) to minimise false negative results so that true cases of heart failure are not missed. At an individual patient level, low specificity is less problematic as individuals will proceed to specialist tests such as echocardiogram to confirm a diagnosis of heart failure but this does have capacity implications for finite echocardiography services.”*

4. Figure 2 is quite cluttered and could potentially be split into two separate plots or otherwise laid out clearer.

Thank you for this suggestion, we have now split the figure into two (Figures 2 and 3) as per your recommendation to show the age-stratified results among people with AF compared to those without.

5. Although often standard text for statistical methods sections, it isn't clear within the context of the paper what p-values are actually being assessed with the "5% threshold to define significance" statement being made. No p-values are reported throughout, presumably as comparing to zero would be largely irrelevant, and the more meaningful 95% confidence intervals are already described elsewhere in methods.

We tend to avoid reporting p-values when working with such a large dataset because this can make some results appear statistically significant when they are not clinically significant. As you say, we feel that 95% confidence intervals are more informative and therefore use these consistently throughout. If the reviewer or editor felt there was a strong case to add p-values in we would be happy to do so, but this would not change the summary of our findings.

Reviewer #2: NT-proBNP is often used to assess suspected heart failure (HF), but levels are elevated in atrial fibrillation (AF), making diagnosis difficult. Atrial fibrillation patients are at higher risk of HF, but an elevated NT-proBNP level in this group does not always indicate HF, leading to diagnostic uncertainty. NT-proBNP has better diagnostic accuracy for HF in patients without atrial fibrillation (AUC = 0.877) than in those with atrial fibrillation (AUC = 0.743). Large cohort (n=155,347), use of UK primary care data linked to secondary care. Real-world data from a representative population over 14 years (2004–2018).

Thank you for reviewing our manuscript and for this helpful summary.

1. Should NT-proBNP thresholds be routinely adjusted for patients with AF in primary care, or should clinicians rely on their clinical judgment?

We now make a more explicit recommendation that the threshold for a positive test NT-proBNP is set at 660pg/mL when considering referring for suspected heart failure in people with AF, compared to 125pg/mL in people without AF as outlined in the Discussion (lines 361 to 363) – see also response to Reviewer 1, comment 2:

*“We suggest a higher threshold of 660pg/mL is considered to account for the fact that AF is associated with raised NT-proBNP levels to help target echocardiogram resources at those most likely to have HF.”*

However, clinical judgement will still be particularly important among people with AF to take account of other factors, such as the patient’s age, current treatment and heart rate as well as the index of suspicion as to a suspected heart failure diagnosis. We have added a note to this effect in the ‘Implications for practice and research’ section (lines 374 to 377):

*“Clinical judgement will remain important when interpreting NT-proBNP results for individual patients with AF to take account of other factors, such as the patient’s age, any concurrent illness, current treatment and heart rate as well as the index of suspicion as to a suspected heart failure diagnosis.”*

2. How might the inclusion of other biomarkers or clinical parameters improve diagnostic accuracy in patients with atrial fibrillation?

We agree that this is an important further research avenue. Whilst the European Society of Cardiology Heart Failure Association provide advice on adjusting NT-proBNP levels on the basis of individual co-morbidities or patient characteristics we are not aware of recommendations that support clinicians in making multiple adjustments based on AF and other factors, such as age or sex. Whilst our analysis goes some way to addressing this research gap by reporting diagnostic accuracy of NT-proBNP in people with and without AF and categorised by age and BMI, we agree there is further work to do in this area. We have added the following comment to the section on ‘Implications for practice and research’ (lines 348 to 351):

*“Future research could explore to what extent NT-proBNP thresholds can simultaneously account for multiple other factors that influence the result including age, BMI, kidney function and current treatment to improve the predictive performance for suspected heart failure.”*

3. How does the lack of left ventricular ejection fraction (LVEF) data limit the interpretation of NT-proBNP performance in different HF subtypes?

We agree that this is to some extent a limitation and is acknowledged as such in the Discussion (lines 329 to 336 – see text below). However, we do think it is important to make clear that in primary care, GPs are recommended to use NT-proBNP to inform who is referred on for further assessment of suspected heart failure and this stage of the referral pathway is independent of the left ventricular ejection fraction (LVEF). Although we could speculate on the diagnostic accuracy of NT-proBNP in people based on the LVEF, this information would not be available to primary care clinicians at the time they are interpreting an NT-proBNP result and therefore we do not feel it would substantially change primary care practice.

*“Coding of HF based on the LVEF is limited within the dataset, precluding an analysis of the relative diagnostic accuracy of NT-proBNP for HF with reduced ejection fraction (HFrEF) or HFpEF. Previous studies have reported that NT-proBNP may be better at detecting patients with HFrEF than HFpEF [23], and that up to one third of patients with HFpEF may have NT-proBNP levels <100pg/mL [24]. However, clinicians referring patients with suspected HF will not know whether a patient has HFrEF or HFpEF at the time of diagnosis, meaning that diagnostic thresholds in the community need to be kept at a uniform level across categories of HF, reflected in our approach to the current analysis.”*

4. Since NT-proBNP values are also influenced by renal function and medications, how could the inclusion of these factors have altered the results?

Impaired renal function is associated with higher levels of NT-proBNP whilst certain medications such as diuretics and RAAS inhibitors are associated with lower levels, in people with and without heart failure. However, we felt that attempting to adjust for these factors on top of the presence of AF, age and BMI was beyond the scope of this analysis. Medication data would have needed to consider multiple different treatments and at different doses, whilst renal function would have required analysis of eGFR and urine albumin: creatinine ratio, both of which are continuous data that would change over follow-up. Such an analysis would have become extremely complex. We do think there is a role for further work that adopts a different analytical approach by seeking to establish NT-proBNP thresholds adjusted for multiple other patient factors, as acknowledged in the section on Implications for practice and research (lines 348 to 351) as follows:

*“Future research could explore to what extent NT-proBNP thresholds can simultaneously account for multiple other factors that influence the result including age, BMI, kidney function and current treatment to improve the predictive performance for suspected heart failure.”*

Reviewer #3: The aim of this study was to assess the diagnostic accuracy of NT-proBNP for HF in a primary care cohort (>155k) of people with AF, overall and by age, sex and BMI.

1. They conclude that NT-proBNP discriminates more accurately for HF among people without AF than with AF. No surprises here.
   Higher natriuretic peptides in AF are well reported, also affected by age, renal function, underlying renal dysfunction, infection etc - also some racial differences too.

Thank you for reviewing the paper and your comments. Although we agree that one might have predicted NT-proBNP discriminates more accurately among people without compared to with AF, we do think that reporting these diagnostic accuracies among a large primary cohort remains important to define these differences and to do so taking account of age, sex and BMI as key covariates.

2. A higher referral threshold could be considered in AF to account for higher median NT-proBNP levels.  Agreed.  This has been advocated in prior papers.

Again, although this approach has been advocated, our analysis reports on the relative diagnostic accuracy of NT-proBNP in people with AF at different thresholds to better inform this decision. To our knowledge, this is by some way the largest diagnostic accuracy study of NT-proBNP for suspected heart failure in people with AF and therefore the results are important in establishing the relative merits of this approach with greater confidence. Comment 2 from Reviewer 6 demonstrates there is still a debate as to how these results should inform practice, with some advocating lower NT-proBNP thresholds among people with AF to avoid missed diagnoses of heart failure.

3. NT-proBNP (and many biomarkers) may be better as a rule out, rather than rule in.  The non-specificity and dynamic changes in levels over time (not just a single 'one off' value) are other limitations of these biomarkers which may simply reflect a sick individual.

Thank you for this reflection. We agree that NT-proBNP is intended as a rule out, rather than rule in, test and have added significant further detail about the role of testing in primary care to the Methods section to make this clear (Methods, lines 128 to 133):

*“NT-proBNP is recommended to assess for heart failure among people with symptoms such as breathlessness, peripheral oedema or fatigue. NT-proBNP is used as a ‘rule out’ test in primary care, because patients without an elevated result are generally not referred on for further assessment of heart failure. Symptomatic patients with an elevated NT-proBNP should be referred for a specialist assessment to confirm or refute the diagnosis of heart failure, including further tests such as an echocardiogram.”*

We recognise that NT-proBNP is a dynamic biomarker but, to our knowledge, repeat or serial NT-proBNP testing is not recommended in any current international guidelines when making an assessment for suspected heart failure. We recognise a range of factors will still need to be taken into account when interpreting NT-proBNP tests and we have added the following summary of this (lines 374 to 377):

*“Clinical judgement will remain important when interpreting NT-proBNP results for individual patients with AF to take account of other factors, such as the patient’s age, any concurrent illness, current treatment and heart rate as well as the index of suspicion as to a suspected heart failure diagnosis.”*

4. The present analysis also does not properly/definitively distinguish HFrEF, HFpEF, HRmrEF etc as LVEF only recorded in 836.

Thank you – this issue was also raised by Reviewer 2. Although we agree this is a limitation (and acknowledge it as such at the start of page 13) we do not believe this categorisation is critical at the time of making an assessment for suspected heart failure in the community at which point NT-proBNP is used to inform who should be referred for further testing rather than informing a definitive heart failure diagnosis. This is discussed in the limitations section of the Discussion (lines 329 to 336) as follows:

*“Coding of HF based on the LVEF is limited within the dataset, precluding an analysis of the relative diagnostic accuracy of NT-proBNP for HF with reduced ejection fraction (HFrEF) or HFpEF. Previous studies have reported that NT-proBNP may be better at detecting patients with HFrEF than HFpEF [23], and that up to one third of patients with HFpEF may have NT-proBNP levels <100pg/mL [24]. However, clinicians referring patients with suspected HF will not know whether a patient has HFrEF or HFpEF at the time of diagnosis, meaning that diagnostic thresholds in the community need to be kept at a uniform level across categories of HF, reflected in our approach to the current analysis.”*

5. The analysis also depends on CPRD, which means that the data are based on subjects who had a blood measurement 'because of something' eg comorbidity or GP suspicion of HF (well, breathlessness) - rather than an unselected population based screening.

Thank you; we agree and had not intended for the results to be interpreted as an analysis of NT-proBNP as a screening tool for heart failure. We have added a much more detailed description of how and why NT-proBNP is used in primary care to the Methods (lines 128 to 133):

*“NT-proBNP is used as a ‘rule out’ test in primary care, because patients without an elevated result are generally not referred on for further assessment of heart failure. Symptomatic patients with an elevated NT-proBNP should be referred for a specialist assessment to confirm or refute the diagnosis of heart failure, including further tests such as an echocardiogram.”*

6. 'Absence of a HF code was assumed to mean absence of a diagnosis.' How accurate is this approach?  Did you test the internal and external validity here?

The code lists to identify cases of heart failure were created by two GPs with experience of heart failure research but it was not possible to validate the accuracy of diagnostic codes within this study. Previous research has reported that codes for heart failure in the primary care clinical record have a high specificity (very likely to represent a true case) but lower sensitivity,^1^ implying some people with heart failure in our dataset would have been inaccurately categorised as not having heart failure. Maintaining a record of patients with heart failure was included as a Quality and Outcomes Framework measure in 2006 and these financial incentives have been shown to improve coding.^2^ Furthermore, we have no reason to suspect miscoding of heart failure would be systematically different between people with or without AF meaning any differences in results would likely be similar between groups. Although this is a limitation in the study, we believe this is outweighed by the benefit of reporting on such a large, longitudinal cohort, as recognised by a number of the reviewers. We have previously published studies using the same primary care database (CPRD) in high impact journals to report on trends in NT-proBNP testing and survival in people with heart failure so there is precedent for this approach.^3,4^

1. Goonasekera MA, Offer A, Karsan W, El-Nayir M, Mallorie AE, Parish S et al. ‘Accuracy of heart failure ascertainment using routinely collected healthcare data: a systematic review and meta-analysis’ *Systematic Reviews* 2024: 13, 79. Doi: 10.1186/s13643-024-02477-5
2. Doran T, Kontopantelis E, Valderas JM, Campbell S, Roland M, Salisbury C and Reeves D. ‘Effect of financial incentives on incentivised and non-incentivised clinical activities: longitudinal analysis of data from the UK Quality and Outcomes Framework’ *BMJ* 201; 342:d3590
3. Roalfe AK, Lay-Flurrie SL, Ordóñez-Mena JM, Goyder CR, Jones NR, Hobbs FDR, Taylor CJ. Long term trends in natriuretic peptide testing for heart failure in UK primary care: a cohort study. Eur Heart J. 2021 Nov 30;43(9):881–91. doi: 10.1093/eurheartj/ehab781. Epub ahead of print. PMID: 34849715; PMCID: PMC8885323.
4. Taylor CJ, Ordóñez-Mena JM, Roalfe AK, Lay-Flurrie S, Jones NR, Marshall T, Hobbs FDR. Trends in survival after a diagnosis of heart failure in the United Kingdom 2000-2017: population based cohort study. BMJ. 2019 Feb 13;364:l223. doi: 10.1136/bmj.l223. Erratum in: BMJ. 2019 Oct 8;367:l5840. doi: 10.1136/bmj.l5840. PMID: 30760447; PMCID: PMC6372921.

Reviewer #4: The manuscript 'NT-proBNP testing for heart failure diagnosis in people with atrial fibrillation: diagnostic accuracy study' addresses an important issue with regards to how to use/interpret NT-proBNP testing in patients with suspicion of HF in the setting of AF. The paper is well written and addresses a difficult aspect of a widely use test in health care overall and primary care in particular. The obvious strength of the current manuscript is the big sample size with more than 150 000 patients included in the analysis. Even though it remains a challenge to decide what the appropriate cut-off for NT-proBNP is in the population in general and AF patients in particular, this study could contribute to a further understanding of the challenges associated with the use of natriuretic peptides in the setting of HF and AF.

Thank you for reviewing the paper, for this helpful summary and for your positive comments.

However, there are some questions remaining after having gone through the manuscript.

1. One important issue is the diagnosis of AF. As I understand it, the patients with AF was categorized to this entity based on the diagnosis of AF in EHR? I suppose that this means that it is sufficient with a diagnosis during the course up to study start, meaning that we have no idea whether the patients were in AF at the time of NT-proBNP testing? If so, I think this is an important limitation when the results are interpreted, where a patients with ongoing fast AF is quite different from a patient with rare episodes of AF and that are in SR during testing. What are the authors thoughts about this? This is an important issue when it comes to implementing the results in clinical practice.

Thank you for raising this important point. Categorisation of AF was based on the presence of a relevant diagnostic code in a patient’s electronic health record at the index date or earlier. It is not possible to tell from routinely collected health record data whether the patient was actually in AF at the time of the NT-proBNP test as this level of detail is difficult to extract from coded entries with any certainty. We also do not have data on patient’s heart rate at the time of NT-proBNP testing, which may also impact upon natriuretic peptide levels in people with or without AF. We have added the following comment in recognition of this to the Limitations section of the Discussion (lines 337 to 342):

*“We did not extract data for cardiovascular medication, heart rate or kidney function as these parameters were not considered critical to the central study design. Furthermore it is not possible to tell whether patients who had been previously diagnosed with AF were actually in AF at the time of their NT-proBNP test. However, each of these factors could be important when interpreting NT-proBNP levels in practice and in determining the optimal threshold to use for suspected heart failure. Further research could involve a dedicated analysis considering these variables.”*

We recognise the need for clinical judgement in interpreting test results given the multiple factors that can impact upon NT-proBNP levels and have added the following comment to the Implications for practice and research (lines 374 to 377) section:

*“Clinical judgement will remain important when interpreting NT-proBNP results for individual patients with AF to take account of other factors, such as the patient’s age, any concurrent illness, current treatment and heart rate as well as the index of suspicion as to a suspected heart failure diagnosis.”*

2. I think the paper could benefit from a figure showing the flow chart of patient inclusion.

Thank you for this suggestion, we have added a flow chart as per your recommendation (see what is now Figure 1).

3. The authors state that kidney disease is a factor that could complicate interpretation of NT-proBNP levels, and in the discussion section list this at one factor to take into account when interpretating the results. Even so, it is stated in the discussion that "....kidney function were not considered critical to the central study design"? Is data regarding kidney function available and if so, have they been tested in the statistical modelling?

Thank you, this issue was also raised by Reviewer 2 (comment 4). We did not extract data on kidney function so this has not been tested. We agree that impaired renal function is associated with higher levels of NT-proBNP whilst certain medications such as diuretics and RAAS inhibitors are associated with lower levels, in people with and without heart failure. However, in this analysis our primary aim was to report on NT-proBNP in people with and without AF and we feel a detailed analysis of how to interpret NT-proBNP in the context of impaired renal function would require a separate, dedicated analysis given it would need to account for changes in renal function over time. When we mention kidney function should be take into account when interpreting NT-proBNP levels in the Discussion, this recommendation is based on prior guidance and not our results.

4. In the methods section, are the codes for the different conditions specified anywhere?

We have added the code lists for heart failure and AF as an appendix (Appendix 1) to the supplementary material.

5. In the results section, it is stated that there were only 836 patients with LVEF recorded, what is the reason for this?

Coding for heart failure in primary care has tended to use codes such as ‘heart failure’ or ‘congestive cardiac failure’ rather than codes based on the left ventricular ejection fraction. This may be due to a number of reasons, including the amount of information that GPs are supplied with from secondary care, the perceived importance of this information to GPs or simply the fact entries into electronic health records are done for the purpose of patient care and not research. The lack of coding by ejection fraction in primary care has been well established in previous research studies.^1^

1. Nakao YM, Nakao K, Nadarajah R, Banerjee A, Fonarow GC, Petrie MC et al. ‘Prognosis, characteristics and provision of care for patients with the unspecified heart failure electronic health record phenotype: a population-based linked cohort study of 95262 individuals’ *eClinicalMedicine* 2023; 63:102164. Doi: 10.1016/j.eclinm.2023.102164
6. Furthermore, in the same area, it is stated that the median LVEF was 48% among people with AF diagnosed with HF compared to 40 % in those not (!) diagnosed with HF? This seem strange.

We apologise that this was a typing error and we were making the comparison between people with AF who were diagnosed with heart failure, compared to people without AF who were diagnosed with heart failure. The text has been corrected and now reads (lines 203 to 206):

*“The left ventricular ejection fraction (LVEF) was only recorded in 836 individuals, 595 of whom were diagnosed with HF, which was too small a proportion to undertake an analysis on this basis. Where reported, the median LVEF was 49% (IQR 35-58%) among people with AF diagnosed with HF compared to 43% (IQR 30-55%) in those without AF who were diagnosed with HF.”*

Reviewer #5: This reviewer can see two versions of the manuscript. However, there is no communication between the authors and reviewers. Thus, this reviewer can only provide comments as for an original submission.

Thank you – we have only submitted this paper once and these comments are our first revision. Perhaps the editorial team could help if further clarification is needed on this point?

The authors utilized real world data from primay and secondary care to look at the dignostic value of NT-proBNP for heart failure in the presence and absence of atrial fibrillation. The primary finding was that NT-pro-BNP behaved poorly in the diagnosis of heart failure in patients with atrial fibrillation. The analysis was well done. The results are well presented.

Thank you for these positive comments and for taking the time to review our manuscript.

1. This reviewer only has several minor comments.
   In the presence of atrial fibrillation, NT-proBNP behaved poor in general, and even poorer with age advancing, not typically in those over 75 years. The authors might make the latter point instead of reporting the poorer results in those over 75 years of age. In fact, the AUC was very similar in those of 65-74 years old.

Thank you, we agree NT-proBNP performs poorly even in people aged 65-74 years, as well as older individuals. We have adjusted this section to read as follows (Results, lines 233 to 239):

*“The presence of AF impacted on the diagnostic accuracy of NT-proBNP to a greater extent than age (Figures 2 and 3) but overall NP testing has very poor discrimination for HF among people with AF who are 65 years or older. For example, among people with AF, the sensitivity and specificity of NT-proBNP at the ESC recommended 125pg/ml threshold was 98.5% (95%CI: 97.8-99.1%) and 18.8% (95%CI: 17.8-19.8%) respectively among people aged 45 to 64 years and 99.0% (95%CI: 98.4-99.4%) and 9.7% (95%CI: 8.9-10.6%) respectively among people aged 65 to 74 years (Table 3).”*

2. The legend inside to Figure 2 is difficult to read. The authors may consider to make some space between those with atrial fibrillation and those without atrial fibrillation similarly as they did for Figure 1.

Thank you for this suggestion. We have split this figure into age-categorised results in people with versus without AF in order to make the figure clearer.

3. The tables are generally big and busy. If possible, the authors may consider to move some of them, for instance, the one between men and women, to supplemental materials, or simplify the contents, for instance the two "total" columns in table 1.

We agree there is a lot of information to present and we apologise if this makes the tables busy to the reader. We are happy to simplify Table 1 by removing the columns displaying total results for those with and without AF. We have also moved the table of diagnostic accuracy results by sex to the supplemental material, as per your suggestion.

Reviewer #6: This study evaluated the diagnostic accuracy of NT-proBNP for heart failure (HF) in patients with and without atrial fibrillation (AF) using the large-scale CPRD GOLD and Aurum databases in UK with 155,347 patients. The authors proposed considering higher AF-specific NT-proBNP thresholds for HF due to elevated baseline levels. The authors addressed a clinically relevant question, i.e. NT-proBNP's utility in HF diagnosis for patients with AF. The large sample size and real-world data linkage are notable strengths.

Thank for taking the time to review the paper and for these positive comments.

However, several methodological and interpretive issues are listed below which warrant discussion: 

1. Study population selection: this study included patients tested for NT-proBNP from the database. This should be clarified whether these corresponded to the suspected HF cases per guidelines, e.g., NICE 2003 (considering that their study started from 2004) recommends natriuretic peptide testing for clinical suspicion using BNP or NT-proBNP where available. Could echocardiograms or other confirmatory tests alone be performed for HF diagnosis? This impacts the interpretation of "diagnostic accuracy," as NT-proBNP is rarely used in isolation.

Thank you, we have added some further clarification on the study population to confirm NT-proBNP is being used in primary care to assess for suspected heart failure, but those patients with an elevated result are then referred on for specialist assessment, including echocardiogram and/or other tests, as deemed necessary by the specialist. As you say, the NT-proBNP alone is not used as a diagnostic test in isolation (Methods, lines 128 to 133):

*“NT-proBNP is recommended to assess for heart failure among people with symptoms such as breathlessness, peripheral oedema or fatigue. NT-proBNP is used as a ‘rule out’ test in primary care, because patients without an elevated result are generally not referred on for further assessment of heart failure. Symptomatic patients with an elevated NT-proBNP should be referred for a specialist assessment to confirm or refute the diagnosis of heart failure, including further tests such as an echocardiogram.”*

Patients who did not have an NT-proBNP and where the diagnosis was made on the basis of other tests such as echocardiogram alone would not have been included in this cohort because the presence of an NT-proBNP test was a study entry criteria. These patients would not therefore impact the results although they do represent an alternative pathway to diagnosis.

2. Statistics for rule-out utility: the emphasis on AUC may be misleading for evaluating a "rule-out biomarker". For exclusion, I believe that NPV and sensitivity are more important. As in Table 2 shows, comparable NPVs for AF vs non-AF patients at 125 pg/mL (97.3% vs. ~98.9%) and 400 pg/mL (94.3% vs. 97.8%). Sensitivity remains high in AF patients (98.8% and 93.2%) at 125 and 400 pg/mL respectively. In my opinion, retaining thresholds for AF patients (e.g., 125 pg/mL) would minimally increase false positives while safeguarding against missed diagnoses, a trade-off favoring safety in real-world practice given AF's around 10% prevalence in the cohort.

We agree that determining the optimal threshold in people with AF is challenging and also that NPV and sensitivity are particularly important when considering a ‘rule-out’ test. However, NICE typically suggest a sensitivity for a primary care rule out test should be at least 80%, which remains true of NT-proBNP at a 660pg/mL threshold among people with AF, whilst the NPV is also >90% at this level. At a threshold of 660pg/mL compared to 125pg/mL would result in 4,535 fewer false positive NT-proBNP results, equivalent to 26.1% of people with AF who would not need referral among this cohort. The cost of this higher threshold would be an additional 538 false negative results (3.1% of people with AF receiving a delayed or missed heart failure diagnosis). As we say in our Discussion (lines 372 to 374):

*“Ultimately the threshold may be determined by healthcare system factors, such as access to echocardiography, where higher NP levels could support a fast-track referral. Implementation studies could help determine the clinical and cost impact of changing the NT-proBNP threshold.”*

3. Outcome definition: the 6-month HF diagnosis endpoint diverges from typical acute diagnostic workflows (where NT-proBNP guides the immediate decisions for further evaluations). Could elevated NT-proBNP in AF have led to earlier HF detection during follow-up, inflating accuracy?

This was a primary care based study from the UK where GPs use NT-proBNP as a test for suspected heart failure. UK guidelines recommend that patients with an elevated NT-proBNP should be referred for specialist assessment and confirmation of the heart failure diagnosis. In addition to adding clarity around the role of NT-proBNP testing in primary care that we outlined in response to your first comment, we have also added additional information regarding the recommended timeframe for specialist assessment for heart failure among people with an elevated NT-proBNP as follows (lines 157 to 160):

*“NICE recommend people with an NT-proBNP ≥2000pg/mL are seen within two weeks of referral and people with an NT-proBNP ≥400pg/mL are seen within six weeks so we report on the proportion of HF diagnoses made within those timeframes.”*

However, in practice these targets are frequently missed so we chose to report on new diagnoses of heart failure within six months of the NT-proBNP test to reflect that some patients would be on a waiting list during this time and may wait some months for the heart failure diagnosis to be confirmed and coded. There is a chance that patients with a relatively low NT-proBNP might not have been seen even after six months, which might have impacted the accuracy of the results and we have added a note to this end to the limitations (Discussion, lines 324 to 328), but we think this would apply to a relatively small number of patients given the generous time window in relation to target waiting times.

*“Some patients may have been referred for suspected heart failure but still not assessed within the six month time window we used leading to erroneous false negative NT-proBNP results. However, given UK guidelines recommend people with a raised NT-proBNP are assessed within six weeks of referral we think this would be unlikely to apply to so many patients it would alter the summary findings.”*

General editorial requests:
(Note: not all will apply to your paper, but please check each item carefully)

*       We ask every co-author listed on the manuscript to fill in a contributing author statement, making sure to declare all competing interests. If any of the co-authors have not filled in the statement, we will remind them to do so when the paper is revised. If all statements are not completed in a timely fashion this could hold up the re-review process. If new competing interests are declared later in the revision process, this may also hold up the submission. Should there be a problem getting one of your co-authors to fill in a statement we will be in contact. Please do not add or remove authors without first discussing this with the handling editor. You can see our competing interests policy here: <http://journals.plos.org/plosmedicine/s/competing-interests>.

Thank you. We have included a competing interests statement in the manuscript with the relevant information.

*       Please upload any figures associated with your paper as individual TIF or EPS files with 300dpi resolution at resubmission; please read our figure guidelines for more information on our requirements: <http://journals.plos.org/plosmedicine/s/figures>. While revising your submission, please upload your figure files to the PACE digital diagnostic tool, <https://pacev2.apexcovantage.com/>. PACE helps ensure that figures meet PLOS requirements. To use PACE, you must first register as a user. Then, login and navigate to the UPLOAD tab, where you will find detailed instructions on how to use the tool. If you encounter any issues or have any questions when using PACE, please email us at [PLOSMedicine@plos.org](mailto:PLOSMedicine@plos.org).

All four of the uploaded figures are TIFF files with 300dpi resolution. We have uploaded these all to the PACE digital diagnostic tool prior to uploading them to the PLOS submission site.

*       Please ensure that the paper adheres to the PLOS Data Availability Policy (see <http://journals.plos.org/plosmedicine/s/data-availability)>, which requires that all data underlying the study's findings be provided in a repository or as Supporting Information. For data residing with a third party, authors are required to provide instructions with contact information (web or email address) for obtaining the data. Please note that a study author cannot be the contact person for the data. PLOS journals do not allow statements supported by "data not shown" or "unpublished results." For such statements, authors must provide supporting data or cite public sources that include it.

We are not all able to share the data used for this study, either via a repository or as supporting information. The study uses data from the Clinical Practice Research Datalink (CPRD), which provides anonymised data for the purpose of the study in question. The license for the data only allows to retain the data for a limited period of time before the data is destroyed and we are not permitted to make the data freely available. We have included the following statement in terms of data availability (lines 412 to 416):

*“Data for this study were obtained on licence from CPRD and cannot be shared due to the contract agreements for the data access. However, equivalent data can be obtained directly from CPRD with relevant ISAC approval. Hospital Episode Statistics and Office for National Statistics data are subject to copyright © (2018) and can be re-used.”*
*       We expect all researchers with submissions to PLOS in which author-generated code underpins the findings in the manuscript to make all author-generated code available without restrictions upon publication of the work. In cases where code is central to the manuscript, we may require the code to be made available as a condition of publication. Authors are responsible for ensuring that the code is reusable and well documented. Please make any custom code available, either as part of your data deposition or as a supplementary file. Please add a sentence to your data availability statement regarding any code used in the study, e.g. "The code used in the analysis is available from Github [URL] and archived in Zenodo [DOI link]" Please review our guidelines at <https://journals.plos.org/plosmedicine/s/materials-software-and-code-sharing> and ensure that your code is shared in a way that follows best practice and facilitates reproducibility and reuse. Because Github depositions can be readily changed or deleted, we encourage you to make a permanent DOI'd copy (e.g. in Zenodo) and provide the URL.

We are happy to upload the code to Github if the manuscript is accepted as part of the publication process. We did not want to delay submitting this response and need a little time to complete that process. We have added the following statement (lines 417 to 418) to which we will add the URL before publication:

*“The code used for this analysis will be uploaded to Github and made freely available at the time of publication.”*

*       The funding statement should include: specific grant numbers, initials of authors who received each award, URLs to sponsors’ websites. Also, please state whether any sponsors or funders (other than the named authors) played any role in study design, data collection and analysis, the decision to publish, or preparation of the manuscript. If they had no role in the research, include this sentence: “The funders had no role in study design, data collection and analysis, decision to publish, or preparation of the manuscript.”

We have updated the funding statement to cover the key requirements, which we include below for ease of reference (lines 391 to 395):

***“Financial disclosure:*** *The original DIAGNOSE-NP study was funded by the National Institute for Health and Care Research (NIHR) Collaboration for Leadership in Applied Health Research and Care (CLAHRC) Oxford (CJT). This work was also supported by the NIHR Community Healthcare Medtech and In Vitro Diagnostics Cooperative (MIC) at Oxford Health NHS Foundation Trust as part of the long-term condition theme (CJT and FDRH).*

*The funders had no role in study design, data collection and analysis, decision to publish, or preparation of the manuscript. The views expressed are those of the authors and not necessarily those of the NHS, the NIHR, or the Department of Health and Social Care.”*

*       Data availability: If the data are not freely available, please describe briefly the ethical, legal, or contractual restriction that prevents you from sharing it. Please also include an appropriate contact (web or email address) for inquiries. Please note that this cannot be a study author.

As outlined above, we are not contractually able to make CPRD data freely available. However, similar data can be obtained via CPRD through their established application process. If key changes are needed to the following data statement (lines 412 to 416) please let us know and we are happy to consider these.

*“Data for this study were obtained on licence from CPRD and cannot be shared due to the contract agreements for the data access. However, equivalent data can be obtained directly from CPRD with relevant ISAC approval. Hospital Episode Statistics and Office for National Statistics data are subject to copyright © (2018) and can be re-used.”*

*       Ethics: Please provide details on consent and ensure to provide the ISAC approval number if available.

Thank you, we have included the following ethics statement in the manuscript (lines 406 to 411):

***“Ethical approval:*** *The DIAGNOSE-NP protocol was approved by the Independent Scientific Advisory Committee (ISAC) of the Medicines and Healthcare products Regulatory Agency (MHRA) (ISAC protocol number: 19_136; available from the authors on request). Ethics approval for observational research using the Clinical Practice Research Datalink (CPRD) with approval from ISAC was granted by a National Research Ethics Service committee (Trent MultiResearch Ethics Committee, reference number: 05/MRE04/87).”*

FORMATTING - GENERAL
*       Abstract: Please structure your abstract using the PLOS Medicine headings (Background, Methods and Findings, Conclusions). Please combine the Methods and Findings sections into one section.

We confirm the abstract is formatted in this manner.

*       At this stage, we ask that you include a short, non-technical Author Summary of your research to make findings accessible to a wide audience that includes both scientists and non-scientists. The Author Summary should immediately follow the Abstract in your revised manuscript. This text is subject to editorial change and should be distinct from the scientific abstract. Ideally each sub-heading should contain 2-3 single sentence, concise bullet points containing the most salient points from your study. In the final bullet point of 'What Do These Findings Mean?', please include the main limitations of the study in non-technical language. Please see our author guidelines for more information: <https://journals.plos.org/plosmedicine/s/revising-your-manuscript#loc-author-summary>.

We have now added the following Author summary (lines 51 to 75):

***“Author summary***

*Why was this study done?*

- *NT-proBNP is a blood test that GPs use to help identify people with suspected heart failure based on their symptoms and risk factors.*
- *People with an elevated NT-proBNP level will be referred to a hospital specialist for further testing to confirm a heart failure diagnosis, whilst people with normal NT-proBNP will be assessed by the GP for alternative causes of their symptoms.*
- *Atrial fibrillation and heart failure frequently co-exist and people with atrial fibrillation typically have higher levels of NT-proBNP but there is uncertainty as to how this should be reflected in threshold levels for referral.*

*What did the researchers do and find?*

- *We used a large database of general practice records to assess how many people who had a NT-proBNP test went on to be diagnosed with heart failure.*
- *The analysis compared the accuracy of NT-proBNP for identifying people with heart failure, comparing between those with and without atrial fibrillation and between sexes, different age groups and categories of body mass index.*
- *We found that NT-proBNP levels are higher in those with atrial fibrillation and it performs less well as a test, so a higher referral threshold may be recommended (660pg/mL) compared to those without atrial fibrillation (125pg/mL).*

*What do these findings mean?*

- *Clinicians will still need to use their clinical judgement when interpreting NT-proBNP but our results could help inform the referral thresholds used in daily practice.*
- *There study’s main limitation is it relies on what has been coded in patient’s electronic health records so cannot tell if people were in atrial fibrillation at the time of NT-propBNP testing or what type of heart failure people went on to develop.”*

*       Please express the main results with 95% CIs as well as p values. When reporting p values please report as p<0.001 and where higher as the exact p value p=0.002, for example. Throughout, suggest reporting statistical information as follows to improve clarity for the reader "22% (95% CI [13%,28%]; p</=)". Please be sure to define all numerical values at first use.

Thank you, we have provided 95% confidence intervals for all results. We tend not to also report p values because we find these can be misleading when working with such a large dataset and small statistically significant results can be wrongly interpreted as being clinically significant. We believe the 95% confidence intervals are therefore more informative.

*       Please include page numbers and line numbers in the manuscript file. Use continuous line numbers (do not restart the numbering on each page).

Thank you, these have been added.

*       Please cite the reference numbers in square brackets. Citations should precede punctuation.

The reference format has been updated to the PLOS style.

FIGURES AND TABLES
*       Please provide titles and legends for all figures and tables (including those in Supporting Information files).

Thank you, these are provided.

*       Please define all abbreviations used in each figure/table (including those in Supporting Information files).

Thank you, we believe we have defined all abbreviations in the figures and tables.

*       Please consider avoiding the use of red and green in order to make your figure more accessible to those with color blindness.

We have updated the colour scheme in the manuscript figures to avoid using red and green together.

SUPPLEMENTARY MATERIAL
*       Please note that supplementary material will be posted as supplied by the authors. Therefore, please amend it according to the relevant comments outlined here.

*       Please cite your Supporting Information as outlined here: <https://journals.plos.org/plosmedicine/s/supporting-information>

We have changed the in-text references to the supplementary tables and figures to refer to these as “S1 table” etc, instead of “Supplemental table 1”. We have also added a list of the different items in the supplementary file after the references within the main manuscript.

REFERENCES
*       PLOS uses the numbered citation (citation-sequence) method and first six authors, et al.

The reference format has been updated to the PLOS style.

*       Please ensure that journal name abbreviations match those found in the National Center for Biotechnology Information (NCBI) databases (<http://www.ncbi.nlm.nih.gov/nlmcatalog/journals>), and are appropriately formatted and capitalised.

Thank you, we believe these are all correctly formatted.

*       Where website addresses are cited, please include the complete URL and specify the date of access (e.g. [accessed: 12/06/2023]).

Thank you, access dates are included with the references.

*       Please also see <https://journals.plos.org/plosmedicine/s/submission-guidelines#loc-references> for further details on reference formatting.

The reference format has been updated to the PLOS style.

STUDY TYPE-SPECIFIC REQUESTS

*       Abstract: Please include the study design, population and setting, number of participants, years during which the study took place (enrollment and follow up), length of follow up, and main outcome measures.

Thank you, the abstract contains these data.

*       For all observational studies, in the manuscript text, please indicate: (1) the specific hypotheses you intended to test, (2) the analytical methods by which you planned to test them, (3) the analyses you actually performed, and (4) when reported analyses differ from those that were planned, transparent explanations for differences that affect the reliability of the study's results. If a reported analysis was performed based on an interesting but unanticipated pattern in the data, please be clear that the analysis was data driven.

Thank you, the manuscript contains this information.

*       Please state in the Methods section whether the study had a prospective protocol or analysis plan. If a prospective analysis plan (from your funding proposal, IRB or other ethics committee submission, study protocol, or other planning document written before analyzing the data) was used in designing the study, please include the relevant document(s) with your revised manuscript as a Supporting Information file to be published alongside your study and cite it in the Methods section. A legend for this file should be included at the end of your manuscript. If no such document exists, please make sure that the Methods section transparently describes when analyses were planned, and when/why any data-driven changes to analyses took place. Changes in the analysis, including those made in response to peer review comments, should be identified as such in the Methods section of the paper, with rationale.

We did not write a specific prospective protocol or analysis plan for this study, which falls within the umbrella of a wider piece of work looking at the role of natriuretic peptides in diagnosing heart failure in primary care. This is stated in the methods.

*       Please ensure that the study is reported according to the STARD guideline (<https://www.equator-network.org/reporting-guidelines/stard/>) and include the completed STARD checklist as Supporting Information. Please add the following statement, or similar, to the Methods: "This study is reported as per the Standards for Reporting of Diagnostic Accuracy (STARD) guideline (S1 Checklist)." When completing the checklist, please use section and paragraph numbers, rather than page numbers.

We have added your suggested sentence referring to the STARD guidelines to the end of the Methods section and included the STARD checklist as a supplemental checklist.

*       Please structure your Abstract according to STARD for Abstracts (<https://www.equator-network.org/reporting-guidelines/stard-abstracts/>).

Thank you, we have structured the abstract according to STARD guidelines.

*       Please structure the Methods section using the following sub-headings: Study design, Participants, Test methods, Analysis.

Thank you, we have structured the Methods using these sub-headings.

*       Please include a diagram to describe the flow of participants through the study (typically figure 1).

We have now included a patient flow diagram as figure 1.
