# Supplementary material for: NT-proBNP testing for heart failure diagnosis in people with atrial fibrillation: A diagnostic accuracy study
Source: PLoS Med. 2025 Oct 30;22(10):e1004550. doi: 10.1371/journal.pmed.1004550 (PMC12574882; doi:10.1371/journal.pmed.1004550)
Supplement: Attachment — Submitted filename: DiagnoseNP in AF PLoS reviewer response 11.8.25.docx [file pmed.1004550.s018.docx]

Thank you for arranging the further review of our paper. We include a point-by-point response to the comments from Reviewer 1 and the Academic Editor below. We have also checked through to ensure our manuscript meets the journal’s formatting requirements. An author summary was included following the initial peer review and we include a statement regarding data availability as requested.

-----------------------------------------------------------

Requests from Editors:

-The suggestion that 26% of people with AF that are currently classified as false positives could be avoided while maintaining the acceptable NPV, merits greater visibility and should be highlighted further in the MS, as well as the abstract and author summary, to emphasize the clinical implications of this finding.

Thank you for this suggestion. We have updated the abstract ‘Methods and Findings’ to read (page 2, lines 47 to 50):

*“Increasing the threshold for a positive test among people with AF from 125pg/mL to 660pg/mL would reduce the number of false positive results by 26.0%, whilst retaining a negative predictive value of 91.5 (95%CI [90.8, 92.1]), albeit with a 10.6% increase in the proportion of those tested with AF having a missed or delayed HF diagnosis.”*

We have updated the author summary to include the following bullet point (page 3, lines 77 to 79):

*“This would reduce the number of false positive NT-proBNP results in people with AF by 26%, whilst retaining the negative predictive value >90%, albeit with a 10% increase in the number of people with AF receiving a false negative result.”*

And we have added further detail to the main text of the manuscript, which now reads as follows (page 12, lines 281 to 291):

*“Within this cohort, 17,403 people tested had pre-existing AF. An NT-proBNP referral threshold of 125pg/mL among people with AF would have resulted in 89.7% (n=15,614) being referred for specialist assessment, with 11,494 false positive results (66.0% of those with AF tested) (Table 2). Setting the threshold at 400pg/mL in people with AF would result in 18.3% (n=3,190) fewer referrals, but with a 7.4% (n=236) increase in the number of people with a missed or delayed diagnosis of HF (S10 table). Increasing the threshold to 660pg/mL in people with AF would mean 29.2% (n=5,073) fewer referrals than the 125pg/ml threshold and a reduction in false positive to 6,959 (40.0% of those with AF tested) (Table 2), a 26% reduction in people with AF receiving a false positive test result. However although the NPV remains >90% at the 660pg/mL threshold among people with AF, using this higher threshold would result in a 10.6% (n=538) increase in the proportion of those tested with AF having a missed or delayed HF diagnosis.”*

Finally, we have also included the following statement at the end of the first paragraph of the Discussion (page 13, lines 309 to 311):

*“Increasing the threshold to 660pg/mL in people with AF would lead to a 26% reduction in false positive results, whilst retaining the NPV >90%, albeit with a 10% increase in false negative results.”*

-Please provide more detail and discussion on potential selection bias. It would be helpful to clarify how this population compares to the real-world population, and a brief discussion of this topic would help the generalizability of the findings.

Thank you for this suggestion. The study cohort is a large, real-world population and includes everybody that had an NT-proBNP test done in primary care within the study period. Although there is likely to have been a degree of selection bias in terms of which patients the GPs chose to order an NT-proBNP test in, this is impossible to quantify and our results do reflect the diagnostic accuracy of NT-proBNP among the patients GPs are selecting for testing. The generalisability of our results to community settings is an important strength of the paper. We have added the following text to the Discussion to address this (page 13, lines 332 to 337):

*“Our study includes everybody who had an NT-proBNP test ordered within a large primary care cohort over a 14 year period, meaning the results are directly applicable to general practice. NT-proBNP tests tend to be ordered by clinicians in primary care for patients with suspected heart failure, but there may be some selection bias in terms of who clinicians selected for testing based on presenting symptoms. Nonetheless, our results inform the interpretation of NT-proBNP among those undergoing testing.”*

-Academic Editor: I would suggest modifying the prevalence in Table 2 to be more specific. It would be useful to include the prevalence of HF according to different cut-offs. If it’s the overall prevalence of HF, it could be listed along with the group title.

We apologise if this was unclear. The prevalence reported is the overall prevalence of heart failure within the populations with and without atrial fibrillation and therefore does not change irrespective of the NT-proBNP cut-off. To make this clearer we have moved this row towards the top of the table, merged the columns to match the group title and updated the row heading to read ‘overall prevalence of heart failure’. The first three rows of Table 2 are now:

|  | **With atrial fibrillation** (n=17,403) | | | | **Without atrial fibrillation** (n=137,944) | | | |
| --- | --- | --- | --- | --- | --- | --- | --- | --- |
| **Overall prevalence of heart failure,**  **% (95% CI)** | 23.9 (23.3-24.6) | | | | 7.6 (7.4-7.7) | | | |
| **NT-proBNP threshold (pg/mL)** | **≥125** | **≥400** | **≥660** | **≥2000** | **≥125** | **≥400** | **≥660** | **≥2000** |

Comments from Reviewers:

Reviewer #1: Reviewer 1:

In my previous review of the article i was of the opinion that it was well written with appropriate methods used, and that remains the case for the re-submission. I believe that the authors have made all reasonable attempts to address my own and other reviewer comments, and that the changes have further improved the article including addressing where there had apparently been some uncertainty in the clinical interpretation.

Thank you for agreeing to review our revised manuscript and for these positive comments.

The only minor comment to address from the responses is for my item #5, where I wasn't suggesting that p-values would need to be added as I agree the confidence intervals are more informative, just that it is odd to state the threshold of significance used for p-values when none are being reported in the paper.

Thank you for this clarification and we apologise for misunderstanding the first time round. We have removed the reference to 5% significance in the methods as we agree it is confusing when we don’t report p-values.

Reviewer #3: No additional comments


Reviewer #5: No further comments.

Thank you to both reviewers for agreeing to check our original and revised submissions.

.

GENERAL EDITORIAL REQUESTS
* Please confirm that your title complies with to PLOS Medicine's style. Your title must be nondeclarative and not a question. It should begin with main concept if possible. "Effect of" should be used only if causality can be inferred, i.e., for an RCT. Please place the study design ("A randomized controlled trial," "A retrospective study," "A modelling study," etc.) in the subtitle (ie, after a colon).

Thank you. We confirm the title complies with PLOS Medicine’s style, including the study design after a colon.

* Please confirm that your abstract complies with our requirements, including format (three sections: Background, Methods and Findings, and Conclusions) and providing all the information relevant to this study type <https://journals.plos.org/plosmedicine/s/submission-guidelines#loc-abstract> 

Thank you. We confirm the abstract complies with PLOS Medicine’s requirements and format.

* Please ensure that the Introduction ends with a clear description of the study question or hypothesis.

The Introduction ends with the stated aim of the study as follows (page 5, lines 117 to 119):

*“The aim of this study was to report the performance of NT-proBNP in diagnosing HF at key thresholds, comparing patients with and without AF, overall and by age, sex and BMI.”*

* Please ensure that all abbreviations are defined at first use throughout the text.

We have checked carefully through and made sure that this is the case.

* Please confirm that all numbers presented in the abstract are present and identical to numbers presented in the main manuscript text.

Thank you, we have checked through the abstract to ensure this is the case.

GENERAL
* Please remove the 'conclusions' subheading from the discussion. Please also remove any other subheadings from the discussion.

Thank you – we have removed all subheadings from the Discussion.

* Statistical reporting: Please revise throughout the manuscript, including tables and figures.
- Please report statistical information as follows to improve clarity for the reader ""22% (95% CI [13,28]; p</=)"".
- Please separate upper and lower bounds with commas instead of hyphens as the latter can be confused with reporting of negative values.
- Please repeat statistical definitions (HR, CI etc.) for each set of parentheses.

Thank you, we have made these changes throughout.

* In the last sentence of the Abstract Methods and Findings section, please describe the main limitation(s) of the study's methodology.

We have added the following sentence to the abstract as requested (page 2, lines 50 to 52):

*“The main limitation of the study is that it relies on routinely collected primary care data and people with an NT-proBNP result <400pg/mL may not have been referred for further assessment, impacting upon the diagnostic accuracy below this threshold.”*

FUNDING STATEMENT
* The funding statement should include: specific grant numbers, initials of authors who received each award, URLs to sponsors’ websites. Also, please state whether any sponsors or funders (other than the named authors) played any role in study design, data collection and analysis, the decision to publish, or preparation of the manuscript. If they had no role in the research, include this sentence: “The funders had no role in study design, data collection and analysis, decision to publish, or preparation of the manuscript.”

Thank you, I have added the specific grant number and URLs although one of the sponsors (the CLAHRC) has closed and been replaced by the Applied Research Collaboration.

* It appears that one or more study authors is affiliated with one or more of the agencies that funded the study. Thus, the statement “The funders had no role in study design, data collection and analysis, decision to publish, or preparation of the manuscript” does not apply. Please revise the Financial Disclosure accordingly, as in "[Author name] is [author's role] at [funding agency]. The funders had no other role in study design…..”  Specifically, this applies to NRJ.

Although Nicholas Jones (NRJ) and Clare Goyder (CG) receive funding for their research time from the NIHR, we do not believe that this affiliation is relevant to the financial disclosure statement. The study was funded by a sub-organisation of the NIHR, called the Collaboration for Leadership in Applied Health Research and Care (CLAHRC) with some support from the NIHR Community Healthcare Medteach and In Vitro Diagnostics Cooperative (MIC). These are separate to the local NIHR funding scheme that supports Clinical Lectureships in Oxford. Although the NIHR provides lectureship funding to NRJ and CG, both are employed by the University of Oxford. We therefore believe it is accurate to state that: “*The funders had no role in study design, data collection and analysis, decision to publish, or preparation of the manuscript. The views expressed are those of the authors and not necessarily those of the NHS, the NIHR, or the Department of Health and Social Care.”*

We do state that “Nicholas R Jones and Clare Goyder are supported by University of Oxford NIHR Academic Clinical Lectureships” (lines 429 to 430) to make clear this relationship.

If the editors feel strongly that they would like us to state this relationship with the NIHR in the financial disclosure statement we are happy to do that.

CODE
* Please state in your data availability statement where the code can be found, with a URL to the GITHUB entry.

We have uploaded the code to a dedicated file on Github (page 18, lines 448 to 449):

***“Code:*** *The code used for this analysis is freely available through Github at: https://github.com/nicholasjones14/DiagnoseNP_AF”*

COMPETING INTERESTS STATEMENT
* All authors must declare their relevant competing interests per the PLOS policy, which can be seen here: <https://journals.plos.org/plosmedicine/s/competing-interests> For authors with ties to industry, please indicate whether any of the interests has a financial stake in the results of the current study.
Thank you, we include a competing interests statement for each author.

DATA AVAILABILITY
* PLOS Medicine requires that the de-identified data underlying the specific results in a published article be made available, without restrictions on access, in a public repository or as Supporting Information at the time of article publication, provided it is legal and ethical to do so. Please see the policy at
<http://journals.plos.org/plosmedicine/s/data-availability> and FAQs at <http://journals.plos.org/plosmedicine/s/data-availability#loc-faqs-for-data-policy>  "

"* The Data Availability Statement (DAS) requires revision. For each data source used in your study:
a) If the data are owned by a third party but freely available upon request, please note this and state the owner of the data set and contact information for data requests (web or email address). Note that a study author cannot be the contact person for the data.
b) If the data are not freely available, please describe briefly the ethical, legal, or contractual restriction that prevents you from sharing it. Please also include an appropriate contact (web or email address) for inquiries (again, this cannot be a study author).

We are legally unable to share these data, but have provided the information outlined above in our Data availability statement as follows (page 18, lines 440 to 447):

***“Data availability statement:*** *Data for this study were obtained on licence from CPRD and cannot be shared due to the contract agreements for the data access. However, equivalent data can be obtained directly from CPRD with relevant ISAC approval. Applications to use CPRD data can be made through the following website:* [*https://www.cprd.com/*](https://www.cprd.com/)*. Enquiries to CPRD can be made at* [*enquiries@cprd.com*](mailto:enquiries@cprd.com)*. We obtained Hospital Episode Statistics and Office for National Statistics data through CPRD linkage and these cannot be shared for the same reason. Hospital Episode Statistics and Office for National Statistics data are subject to copyright © (2018) and can be re-used with the permission of The Health and Social Care Information Centre. All rights reserved.”*

ETHICS AND CONSENT
*Thank you for including an ethics statement. However this is not, as mentioned in the metadata, present in the Methods section’. Please ensure the metadata correctly states where the ethics statement can be found.

* Please specify whether informed consent was written or oral. Please ensure that the research complies with the PLOS policy in full: <https://journals.plos.org/plosmedicine/s/human-subjects-research#loc-patient-privacy-and-informed-consent-for-publicationNT>

Thank you. We have included a statement regarding ethics with the study manuscript. Individual patients within CPRD do not provide explicit individual consent for research but can choose to opt out if they so choose (page 5, lines 129 to 133):

*“Ethics approval for observational research using CPRD has previously been granted by a National Research Ethics Service committee (Trent MultiResearch Ethics Committee, reference number: 05/MRE04/87). Individual patients in the dataset do not provide explicit consent for their data to be used for research purposes but can choose to opt out of research through the national data opt out process, as outlined at https://digital.nhs.uk/services/national-data-opt-out.”*

FIGURES
* Please number the ‘central illustration’ so it is clear where in the manuscript this fits.

We have renamed the central illustration as Figure 2.

DIAGNOSTIC TESTS
*Did your study have a prospective protocol or analysis plan? Please state this (either way) early in the Methods section.

a) If a prospective analysis plan (from your funding proposal, IRB or other ethics committee submission, study protocol, or other planning document written before analyzing the data) was used in designing the study, please include the relevant prospectively written document with your revised manuscript as a Supporting Information file to be published alongside your study, and cite it in the Methods section. A legend for this file should be included at the end of your manuscript.

b) If no such document exists, please make sure that the Methods section transparently describes when analyses were planned, and when/why any data-driven changes to analyses took place.

c) In either case, changes in the analysis-- including those made in response to peer review comments-- should be identified as such in the Methods section of the paper, with rationale.

We did not publish a prospective protocol; this is acknowledged in the Methods (page 5, lines 124 to 125):

*“An application was submitted to CPRD to access the data but we did not pre-publish a study protocol.”*

The ISAC application approved by CPRD does include an analysis plan for the wider DIAGNOSE-NP study but this is not in a format that we can share online. A summary of the study is listed on the CPRD website at <https://www.cprd.com/approved-studies/natriuretic-peptide-testing-diagnosing-heart-failure-primary-care-diagnose-np>.

The deviation from the ISAC protocol for this analysis was to report on an NTproBNP threshold of 660pg/mL in addition to 125, 400 and 2000pg/mL. This reflects the fact 660pg/mL is recommended in European guidelines for diagnosing heart failure with preserved ejection fraction among people with atrial fibrillation. This threshold was decided before any analysis was undertaken. No additional post-hoc analyses were undertaken, including none required in response to peer review.

We have slightly amended the wording in the analysis plan to be explicit the 660pg/mL was different to the previous analyses we have undertaken with this data (page 7, lines 170 to 172):

*“In contrast to our previous analyses of this dataset, within this study we include an analysis at a threshold of 660pg/mL because this reflects the value the ESC recommend as a major criterion for diagnosing HFpEF among people with AF [1-3, 5]”*

* Address the extent to which the study population is representative of the population of interest

Thank you, we believe this is addressed by the following description in the Methods (page 5, lines 125 to 126):

*“The combined databases contain the patient records of >15% of the UK population and have been shown to be representative of the wider population [12].”*

As well as the additional text in the Discussion (page 13, lines 332 to 337):

*“Our study includes everybody who had an NT-proBNP test ordered within a large primary care cohort over a 14 year period, meaning the results are directly applicable to general practice. NT-proBNP tests tend to be ordered by clinicians in primary care for patients with suspected heart failure, but there may be some selection bias in terms of who clinicians selected for testing based on presenting symptoms. Nonetheless, our results inform the interpretation of NT-proBNP among those undergoing testing.”*
